# Tautomer-Specific Deacylation and Ω-Loop Flexibility Explain the Carbapenem-Hydrolyzing Broad-Spectrum Activity of the KPC-2 β-Lactamase

**DOI:** 10.1021/jacs.2c12123

**Published:** 2023-03-27

**Authors:** Catherine L. Tooke, Philip Hinchliffe, Michael Beer, Kirill Zinovjev, Charlotte K. Colenso, Christopher J. Schofield, Adrian J. Mulholland, James Spencer

**Affiliations:** † School of Cellular and Molecular Medicine, Biomedical Sciences Building, University Walk, University of Bristol, Bristol BS8 1TD, United Kingdom; ‡ Centre for Computational Chemistry, School of Chemistry, Cantock’s Close, University of Bristol, Bristol BS8 1TS, United Kingdom; § School of Biochemistry, Biomedical Sciences Building, University Walk, University of Bristol, Bristol BS8 1TD, United Kingdom; ∥ Departamento de Química Física, Universitat de València, Burjassot 46100, Comunitat Valenciana, Spain; ⊥ Chemistry Research Laboratory, Department of Chemistry and the Ineos Oxford Institute for Antimicrobial Research, Mansfield Road, University of Oxford, Oxford OX1 3TA United Kingdom

## Abstract

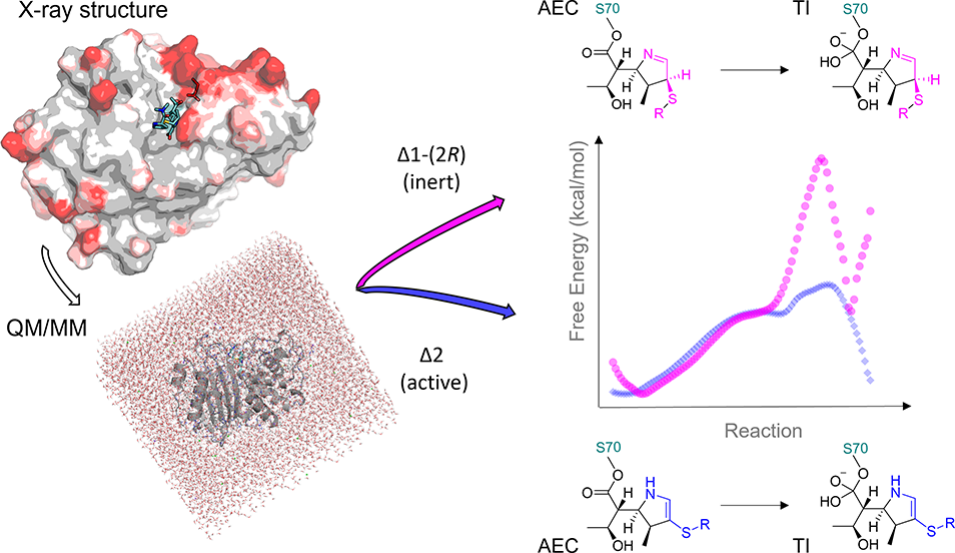

KPC-2 (*Klebsiella pneumoniae* carbapenemase-2) is a globally disseminated serine-β-lactamase (SBL) responsible for extensive β-lactam antibiotic resistance in Gram-negative pathogens. SBLs inactivate β-lactams via a mechanism involving a hydrolytically labile covalent acyl-enzyme intermediate. Carbapenems, the most potent β-lactams, evade the activity of many SBLs by forming long-lived inhibitory acyl-enzymes; however, carbapenemases such as KPC-2 efficiently deacylate carbapenem acyl-enzymes. We present high-resolution (1.25–1.4 Å) crystal structures of KPC-2 acyl-enzymes with representative penicillins (ampicillin), cephalosporins (cefalothin), and carbapenems (imipenem, meropenem, and ertapenem) obtained utilizing an isosteric deacylation-deficient mutant (E166Q). The mobility of the Ω-loop (residues 165–170) negatively correlates with antibiotic turnover rates (*k*_cat_), highlighting the role of this region in positioning catalytic residues for efficient hydrolysis of different β-lactams. Carbapenem-derived acyl-enzyme structures reveal the predominance of the Δ1-(2*R*) imine rather than the Δ2 enamine tautomer. Quantum mechanics/molecular mechanics molecular dynamics simulations of KPC-2:meropenem acyl-enzyme deacylation used an adaptive string method to differentiate the reactivity of the two isomers. These identify the Δ1-(2*R*) isomer as having a significantly (7 kcal/mol) higher barrier than the Δ2 tautomer for the (rate-determining) formation of the tetrahedral deacylation intermediate. Deacylation is therefore likely to proceed predominantly from the Δ2, rather than the Δ1-(2*R*) acyl-enzyme, facilitated by tautomer-specific differences in hydrogen-bonding networks involving the carbapenem C-3 carboxylate and the deacylating water and stabilization by protonated N-4, accumulating a negative charge on the Δ2 enamine-derived oxyanion. Taken together, our data show how the flexible Ω-loop helps confer broad-spectrum activity upon KPC-2, while carbapenemase activity stems from efficient deacylation of the Δ2-enamine acyl-enzyme tautomer.

## Introduction

**Figure 1:**
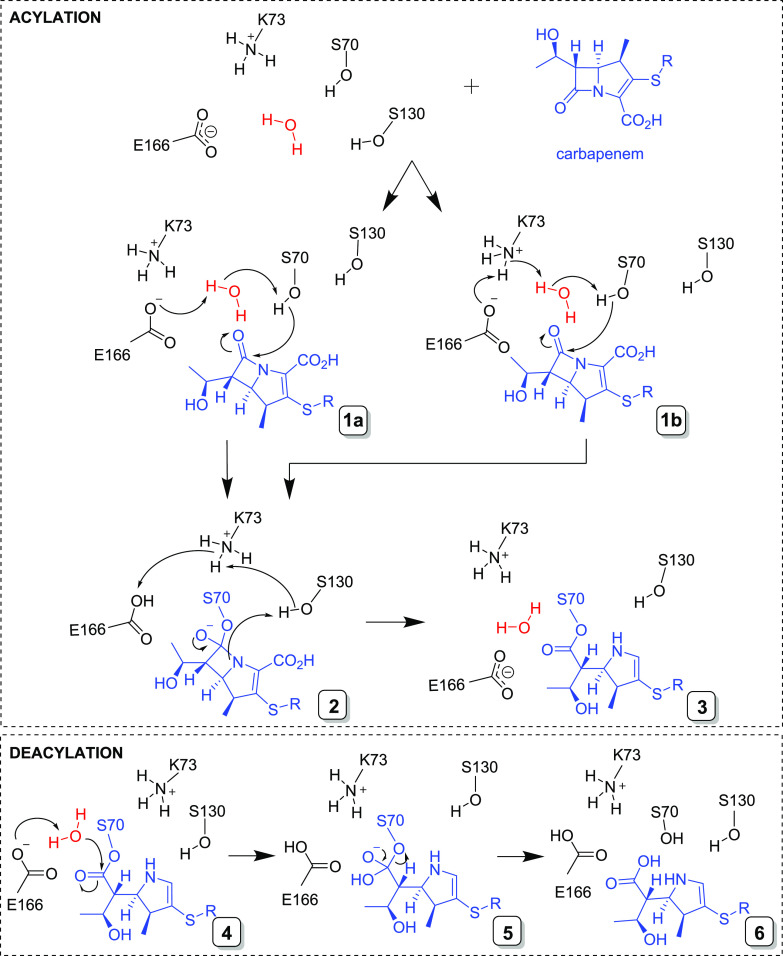
Reaction of carbapenems with class A serine β-lactamases. Top: acylation; binding of a 1β-methyl carbapenem (blue) to the class A SBL active site is followed by acylation.^6^ Ser70 is activated via an active site water (red) with either Glu166^7^ (**1a**) and/or Lys73 (**1b**) acting as a general base, and a subsequent nucleophilic attack on the C-7 carbonyl forms a tetrahedral intermediate (**2**), which reacts to give the covalent acyl-enzyme complex (**3**; for clarity, only the Δ2-enamine is shown). Bottom: deacylation;^8^ proton transfer from the deacylating water molecule (red) to Glu166 enables a nucleophilic attack on the C-7 carbonyl of the acyl-enzyme complex (**4**), resulting in a second tetrahedral intermediate (**5**) that collapses to give the hydrolyzed product (**6**).

β-Lactams are the most prescribed antibiotics worldwide for the treatment of serious healthcare-associated infections (HAIs) caused by Gram-negative bacterial pathogens.^1,2^ Many aspects of healthcare are threatened by increasing resistance to these antibiotics. Approximately 2.8 million antibiotic-resistant infections occur each year in the USA,^3^ and 1.27 million deaths worldwide are attributed to bacterial resistance to antibiotics; these numbers are growing.^4^ The most important mechanism of β-lactam resistance in Gram-negative bacteria is the production and activity of serine β-lactamases (SBLs), a diverse class of hydrolytic enzymes that inactivate all classes of β-lactam antibiotics.^5^ SBLs are split into three (Ambler) classes, namely, A, C, and D, based on sequence and mechanism.^5^ In all three classes, SBL-catalyzed β-lactam hydrolysis proceeds through two main steps: attack of the nucleophilic serine on the β-lactam carbonyl carbon with cleavage of the β-lactam amide bond to form a covalent acyl-enzyme intermediate (acylation)^6,7^ followed by deacylation through the attack of an activated water molecule on the acyl-enzyme carbonyl to release the inactivated hydrolyzed antibiotic^8^ (Figure 1).

**Figure 2:**
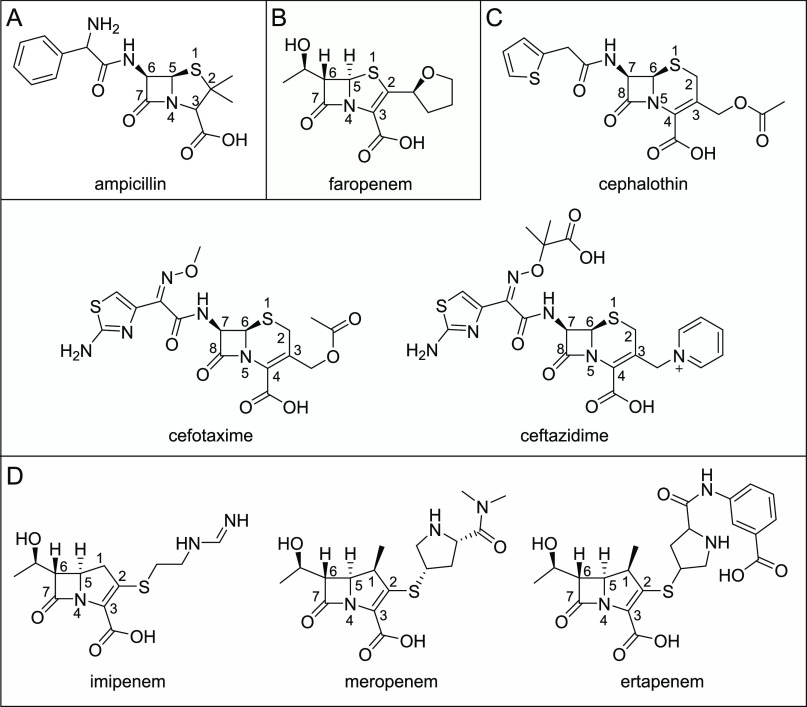
Structures of β-lactam antibiotics studied here. (A) Ampicillin (penicillin/penam). (B) Faropenem (penem). (C) Cephalothin, cefotaxime, and ceftazidime (cephalosporins). (D) Imipenem, meropenem, and ertapenem (carbapenems).

**Figure 3:**
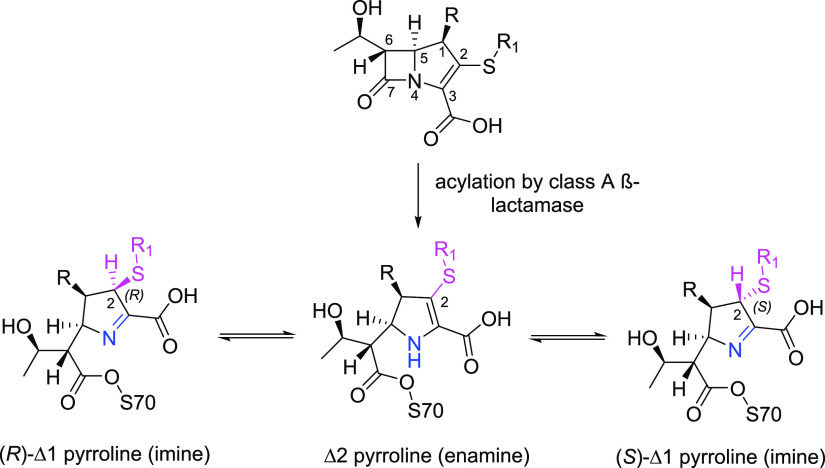
Carbapenem tautomerization in acyl-enzyme complexes of class A β-lactamases.

Carbapenems are potent β-lactams used for treatment of severe HAIs caused by opportunistic Gram-negative species. Carbapenems contain a five-membered pyrroline ring fused to a four-membered β-lactam ring and were first discovered as natural products, such as thienamycin, but those used clinically are produced by total synthesis.^9−11^ Their pyrroline ring differentiates them from the penicillins and cephalosporins, which contain a β-lactam rings fused to a thiazolidine or a dihydrothiazine ring, respectively. Furthermore, the 6α-hydroxyethyl carbapenem substituent, which differs from the typically larger β-substituent groups attached to C-6/C-7 in penicillin and cephalosporin antibiotics (Figure 2), is thought to protect carbapenems against hydrolysis by most β-lactamases.^9^ Indeed, carbapenems were originally discovered as both antimicrobials and β-lactamase inhibitors and are known to resist deacylation by many SBLs (e.g., the class A TEM, SHV, and CTX-M enzymes) by forming long-lived acyl–enzyme complexes.^5^ The development of carbapenems to improve pharmacological activity and potency involved the addition of a 1β-methyl group (to protect against hydrolysis by human dehydropeptidase-I^9^) and alternative substituents at the (sp^2^-hybridized) C-2 position (R1 groups; Figure 3).

The presence of a double bond in the carbapenem pyrroline ring permits tautomerization of the acyl-enzyme complex formed between Δ1-pyrroline and Δ2-pyrroline forms (Figure 3). Migration of the double bond from C-2=C-3 (Δ2, enamine) to C-3=N (Δ1, imine) with associated protonation at C-2 gives rise to the possibility of alternative enamine and imine tautomers in both acyl-enzymes and hydrolysis products with Δ1 in either the 2*R* or 2*S* configurations. In the class A SBL SHV-1, which lacks carbapenemase activity, Raman spectroscopy of the acyl-enzyme indicated a rapid deacylation of a Δ2 enamine and prolonged stability of Δ1, leading to the suggestion that accumulation of the latter acyl-enzyme is the basis for a poor turnover.^12^ In this and similar enzymes, deacylation of carbapenem acyl-enzymes is believed to be retarded by interaction of the 6α-hydroxyethyl group with the deacylating water molecule, either reducing its nucleophilicity or perturbing its position.^13−15^

Since the introduction of carbapenems in the early 1980s, β-lactamases that efficiently inactivate these antibiotics (carbapenemases) have emerged.^16^ These include carbapenem-hydrolyzing SBLs that now significantly challenge the clinical efficacy of all members of this potent β-lactam antibiotic class.^16^ Such resistance is now common with carbapenem-resistant *Acinetobacter baumannii*, *Pseudomonas aeruginosa*, and *Enterobacterales*, which are all now listed as WHO-priority pathogens.^17^ One such carbapenem-hydrolyzing enzyme, *Klebsiella pneumoniae* carbapenemase-2 (KPC-2), is a class A SBL first identified from a carbapenem-resistant *Klebsiella pneumoniae* isolate from North Carolina.^18^ KPC-2 is now present worldwide in numerous pathogenic Gram-negative organisms.^19^ KPC-2 is described as a “versatile β-lactamase” because it efficiently hydrolyzes most penicillins, cephalosporins, and carbapenems and resists the action of inhibitors based on the β-lactam scaffold.^5,9,16,20^

In KPC-2 and other class A SBLs, the active site (containing the nucleophilic Ser70) is bordered by the flexible Ω-loop where the general base for deacylation (Glu166) resides.^5,7,13^ In crystal structures of unliganded KPC-2, Ser70 and Glu166 participate in conserved H-bond networks that also involve Lys73, Ser130, Asn170, and an active-site water molecule apparently positioned for the deacylation reaction (Figure 1 and Figure S1). We recently showed how plasticity in and around the KPC-2 active site enables the accommodation of more “bulky” antibiotics such as the expanded-spectrum oxyimino-cephalosporin ceftazidime, although this adversely affects turnover rates due to the displacement of the Ω-loop and consequent imperfect positioning of Glu166.^13^

Quantum mechanics/molecular mechanics (QM/MM) simulations of deacylation can be used as a “computational assay” to assess the ability of class A β-lactamases to hydrolyze carbapenems and discriminate between carbapenemases and non-carbapenemases on the basis of calculated reaction barriers.^21^ Such simulations identify determinants of carbapenem turnover (for example, in carbapenem-hydrolyzing enzymes, an interaction with Asn132 positions the 6α-hydroxyethyl substituent away from a direct interaction with the deacylating water^14,22^) but require high-resolution structures of acyl-enzyme complexes, which remain limited. As a consequence, a comprehensive description of the basis for the different activities of class A β-lactamases toward carbapenems remains to be achieved. In particular, the basis for the carbapenem-hydrolyzing activity of KPC and related enzymes, which distinguishes them from the great majority of class A β-lactamases; and how the KPC-2 active site ensures efficient carbapenem deacylation, are poorly understood.^5^

Here, we present crystal structures of carbapenem acyl-enzyme complexes of the deacylation-deficient KPC-2 Glu166Gln mutant (KPC-2^E166Q^), which unexpectedly contain the Δ1, rather than the Δ2, tautomer. Using these structures, we explored the dynamics of the acyl-enzyme complex of the carbapenem meropenem with wild-type KPC-2 in 1.5 μs of MM molecular dynamics (MD) simulations and applied QM/MM calculations to investigate the deacylation reaction of both the meropenem Δ1 and Δ2 tautomers using the adaptive string method.^23^ The results indicate that a key factor underlying the efficient hydrolysis of multiple β-lactam classes by KPC-2 is the ability of the Ω-loop to enable efficient positioning of the deacylating water (DW) in the respective acyl-enzymes. The simulations show a significant difference in reactivity between the Δ1 and Δ2 acyl-enzymes, Furthermore, efficient deacylation of carbapenem acyl-enzymes in the Δ2 enamine tautomer is enabled by hydrogen-bonding networks involving the C-3 carboxylate and 6α-hydroxyethyl groups and DW. These data identify the basis for the broad spectrum of activity of KPC-2 and how this enzyme and, by implication, other class A carbapenemases efficiently deacylate carbapenem acyl-enzymes. Our results will inform the design of both new generations of β-lactamase inhibitors and of new β-lactams that are able to evade the versatile broad-spectrum activity of KPC-2.

## Methods

### Enzyme Assays

All enzyme assays were followed at 25 °C in 10 mM HEPES pH 7.5, 150 mM NaCl in Greiner half-area 96-well plates, and a Tecan Infinite 200 pro microplate reader. Steady-state kinetic parameters were calculated by measuring β-lactam antibiotic hydrolysis (ampicillin Δ235 ε = −900, cefalothin Δ262 ε = −7660, imipenem Δ299 ε = −9000, meropenem Δ297 ε = −6500, and ertapenem Δ295 ε = −7112) with 10 nM KPC-2 with 50 ug/mL BSA. Initial rates (*V*_0_) of β-lactam hydrolysis were plotted against the concentration of the antibiotic, and kinetic parameters were calculated and analyzed by least-squares fitting to the Michaelis–Menten equation in GraphPad Prism 6 (GraphPad, La Jolla, California, USA; www.graphpad.com).

#### Protein Crystallization, Antibiotic Complex Generation, and X-ray Diffraction Data Collection

KPC-2 and KPC-2^E166Q^ were produced in recombinant *E. coli* using the pET28a T7 expression vector and were purified and crystallized as described previously.^13^ KPC-2^E166Q^ crystals were soaked with solutions of 10 mM ampicillin (Sigma), 15 mM cefalothin (Sigma), 15 mM imipenem (Sigma), 30 mM meropenem (Sigma), and 30 mM ertapenem (MedChemExpress, USA) dissolved in mother liquor (2.0 M ammonium sulfate, 5% ethanol) supplemented with 20–30% glycerol. Crystals were soaked from 5 min to several hours. The datasets presented here were of the best quality of those obtained (determined by the resolution, ligand occupancy, and real-space correlation coefficient (RSCC) alongside overall data collection and refinement statistics) and were collected after soaking crystals for 2.5 h (KPC-2^E166Q^:ampicillin and KPC-2^E166Q^:cefalothin, KPC-2^E166Q^:imipenem, and KPC2^E166Q^:meropenem) and 3 h (KPC2^E166Q^:ertapenem). Diffraction data were collected at the ALBA synchrotron beamline BL13 XALOC. Images were indexed and integrated using XDS^24^ and subsequently scaled in AIMLESS^25^ (CCP4 suite).^26^ Crystallographic phases were calculated in Phaser^26,27^ using PDB 6Z21(13) (crystal structure of apo KPC-2^E166Q^) as a molecular replacement solution. Initial refinements in REFMAC5^28^ confirmed the *F*_o_ – *F*_c_ electron density to be consistent with the ligand bound at the active site prior to further rounds of refinement in Phenix.refine^29^ and manual model building in Coot.^26,30^ Geometry restraints for antibiotic-derived ligands were calculated using eLBOW in Phenix,^29^ and omit maps were generated in Phenix^29^ from the final model in the absence of the antibiotic. Ligand occupancies were initially manually assigned based upon visual inspection of the electron density and subsequently refined in Phenix^29^ with at least 10 rounds of refinement. Figures were generated in Pymol.^31^

### Molecular Dynamics Simulations

Crystal structures of KPC-2 (PDB 5UL8),^32^ KPC-2^E166Q^ (PDB 6Z21),^13^ and KPC-2^E166Q^:meropenem (solved here) were used as starting structures for molecular simulations. For simulations of the wild-type KPC-2, Gln166 was edited to Glu166^13^ in Coot.^30,33^ The Δ2 meropenem-derived acyl-enzyme complex was modeled in Coot into KPC-2 using the electron density of the Δ1-(2*R*) meropenem-derived acyl-enzyme complex as a guide. The Δ1-(2*R*) complex of KPC-2:meropenem was chosen as the most representative carbapenem complex structure for simulation here because all meropenem atoms were well defined by the experimental electron density and meropenem has both a smaller C-2 substituent than ertapenem and contains a C-1 methyl group (absent from imipenem). In addition, the Δ1-(2*R*) complex was the most frequently observed carbapenem tautomer crystallographically and consistently refined with the highest occupancy. The resulting protein coordinates (KPC-2^E166Q^, KPC-2, KPC-2^E166Q^:Δ1-(2*R*)-meropenem and KPC-2:Δ1-(2*R*)-meropenem, and KPC-2^E166Q^:Δ2-meropenem and KPC-2:Δ2-meropenem) were parameterized for molecular simulations. All crystallographically observed water molecules were included in the simulations. The protonation states of titratable residues were determined using the PropKa 3.1 server.^34^ Hydrogens were added in tleap (AMBER16^35^), and the systems were solvated using a 10 Å waterbox (TIP3P) with overall charges neutralized by addition of Na^+^ or Cl^–^ ions replacing bulk water molecules. Atomic charges for the meropenem (Δ1-(2*R*)/Δ2) acyl-enzymes were generated using restrained electrostatic potential (RESP) fitting as implemented in the RED server.^36^ All structures underwent a standard energy minimization (600 steps of steepest descent and 600 steps of conjugate gradient), heating (25–298 K in 20 ps), and equilibration by MM MD (1 ns) protocol. The structures were then simulated using MM MD in the AMBER16 simulation package using the ff14SB MM force field for proteins, the TIP3P-Ew water model, and the general AMBER force field (GAFF) for ligands. All six protein systems were simulated in triplicate for 100 ns; KPC-2, KPC-2:Δ1-(2*R*)-meropenem, and KPC-2:Δ2-meropenem were further simulated in triplicate runs of 500 ns each (1.5 μs in total). RMSD, RMSF, clustering, distance, and dihedral analyses were performed in CPPTRAJ in AMBER16. RMSD calculations were performed using the first frame (1 ps) as the reference.

#### QM/MM Calculations of Carbapenem Deacylation (Tetrahedral Intermediate Formation)

An implementation of the adaptive string method (ASM) with AMBER18^37^ was used to calculate the minimum free energy path (MFEP).^23^ Two collective variables were chosen to monitor the reaction progress: the distance between the transferred proton of the DW and the Glu166 side chain (collective variable 1: *rx* = *d*(OεGlu166–HDW)) and the distance between the oxygen of the DW and meropenem C-7 carbon (collective variable 2: *ry* = *d*(C-7meropenem–ODW)) (Figure S2). The ASM applies an on-the-fly string method for defining the position of the minimum free energy pathway (MFEP). The position of the string nodes (after sufficient string position optimization) is then used to define the reaction coordinate (RC), and umbrella sampling (US) windows are used to calculate the potential of mean force (PMF) of the proposed reaction pathway. The ASM differs from conventional umbrella sampling (US) in that it does not use user-specified RC and US windows, providing a flexible description of the reaction and allowing a more accurate identification of, for example, TS structures (free energy maxima) and associated barriers.^23^ For simplicity, here we refer to this method as the ASM and conventional umbrella sampling as US, despite both methods employing umbrella sampling to obtain data for PMF calculation.

Acyl-enzyme complex and tetrahedral intermediate structures for string method calculations were established through US calculations of the minimum free energy path (MFEP) using the same reaction coordinates (Note S1), as previously described.^15,21^ The QM calculations used the approximate density functional theory (DFT) self-consistent-charge density-function tight-binding (SCC-DFTB) method^38,39^ with the ff14SB MM force field^40^ for the protein, the TIP3P-Ew for the DW water model, and the General AMBER force field (GAFF)^37^ for those parts of meropenem not included in the QM region.^15,21^ While the SCC-DFTB2 method is known to underestimate barrier heights for deacylation reactions (as shown by comparison to experimentally derived values), we have previously demonstrated that the QM/MM calculations at this QM level give relative barrier heights that are predictive of carbapenemase activity across class A enzymes.^15,21^ The QM region (total charge of −2e) comprised the deacylating water (DW), the side chains of Ser70 and Glu166, and the carbapenem scaffold (Figure S2)^15^ as tested in previous simulations.^15,21^ The string was composed of 28 nodes. Each initial starting structure (acyl-enzyme complex) was obtained after 300 ps of QM/MM MD and the end structure (tetrahedral intermediate), by 20 ps sampling in an umbrella sampling simulation at the desired reaction coordinate. No nodes were fixed to allow the system to relax, and no additional restraints were placed on any atoms.

During the string optimization phase of the ASM simulations, string convergence was monitored by calculating the RMSD of the string at each step compared to all the previous states of the string. In all cases, convergence of the string was reached by 50 ps and converged parameters for the string were obtained by averaging over 10,000 steps from when the string was judged to have reached convergence. Sixty picoseconds of umbrella sampling per string node was then run with these parameters and the potential of mean force (PMF) calculated with umbrella integration.^41^ This procedure was repeated three times for the Δ1-(2*R*) and Δ2 tautomers using different initial and final structures (acyl-enzyme complex and tetrahedral intermediate structures obtained from independent simulations as described above) in each repeat. The MFEP was analyzed over a set of 28 trajectories, one for each node. TS structures were taken from frames with a free energy within 0.05 kcal/mol of the calculated barrier. Each trajectory was analyzed using CPPTRAJ from AmberTools16.^42^

### DFT Calculations on Meropenem Acyl-Enzymes

Calculations on small model complexes using high-level DFT (B3LYP-GD3BJ/6-31 + G(d,p), that is^43^ the B3LYP functional^44^ with additional dispersion corrections^45^ using Grimme’s D3 correction with Becke-Johnson damping^46^ were performed on structures from QM/MM MD simulations of the Δ1-(2*R*) and Δ2 acyl-enzymes above. These were first energy-minimized at the SCC-DFTB2/MM level.^39^ To account for solvent effects, the conductor-like polarized continuum model (CPCM)^47,48^ was used. A dielectric constant of ε = 78.4 was used when the model contained a ligand covalently attached to Ser70 only, representing a minimal-reacting system in solvent. All atoms of Ser70 were included as the backbone amide is known to interact with meropenem. For a larger active-site model, additionally including the Cα and side-chain atoms of the residues Lys73, Ser130, Asn132, Glu166, Asn170, Thr216, and Thr235 and all atoms of Thr237 (because its backbone amide contributes to the oxyanion hole) and the deacylating water, a dielectric constant of ε = 4 was used to represent the protein environment approximately as is commonly used in DFT calculations on enzyme active sites.^49,50^ All Cα atoms were frozen for DFT geometry optimization calculations to maintain the general architecture of the active site model, while no atoms were frozen in the Ser70–meropenem-only model. The set-up for these calculations was completed in GaussView 6.1.1,^51^ while the calculations were run using Gaussian 16.^51^

## Results

### Kinetic and Structural Analysis of Antibiotic Hydrolysis by KPC-2

KPC-2 is a carbapenemase that also hydrolyzes a wide variety of other β-lactams, including penicillins and cephalosporins, as demonstrated by steady-state kinetic data (Table 1).^52−55^ KPC-2 efficiently catalyzes the hydrolysis of the three carbapenems tested here, although the *K*_M_ value for the hydrolysis of imipenem (which contains a 1β-hydrogen rather than a 1β-methyl group) is an order of magnitude larger than for the 1β-methyl-substituted carbapenems, ertapenem, and meropenem (*K*_M_ values of 72, 7.1, and 8.6 μM, respectively). The *K*_M_ values also increase for β-lactam antibiotics with larger C-6/C-7 groups in the β-orientation (i.e., cephalosporins and penicillins) compared to the relatively small 6-hydroxyethyl group in the α-orientation that is present in carbapenems and penems (Table 1).

**Table 1:** KPC-2 Steady-State Kinetics^a^

antibiotic	class	*k*_cat_ (s^–1^)	*K*_M_ (μM)	*k*_cat_/*K*_M_ (μM^–1^ s^–1^)	*K*_i_ (μM)	ref
ampicillin	penicillin	82 (3.9)	270 (45)	0.30 (0.052)		this work
cefalothin	cephalosporin	110 (2.8)	42 (3.4)	2.7 (0.23)		this work
cefotaxime	cephalosporin	76 (6.6)	200 (29)	0.38 (0.064)		(13)
ceftazidime	cephalosporin	1.9 (0.12)	530 (69)	0.0035 (5.2 x 10^–4^)		(13)
faropenem	penem	3.71 (0.21)	16.6 (3.84)	0.22 (0.053)	44.1 (25.0)	(56)
imipenem	carbapenem	22 (0.43)	72 (3.2)	0.31 (0.015)		this work
meropenem	carbapenem	21 (0.12)	7.1 (1.0)	3.00 (0.42)	500 (140)	this work
ertapenem	carbapenem	8.0 (0.26)	8.6 (0.90)	0.92 (0.10)	710 (100)	this work

a Standard errors are in parentheses.

**Figure 4:**
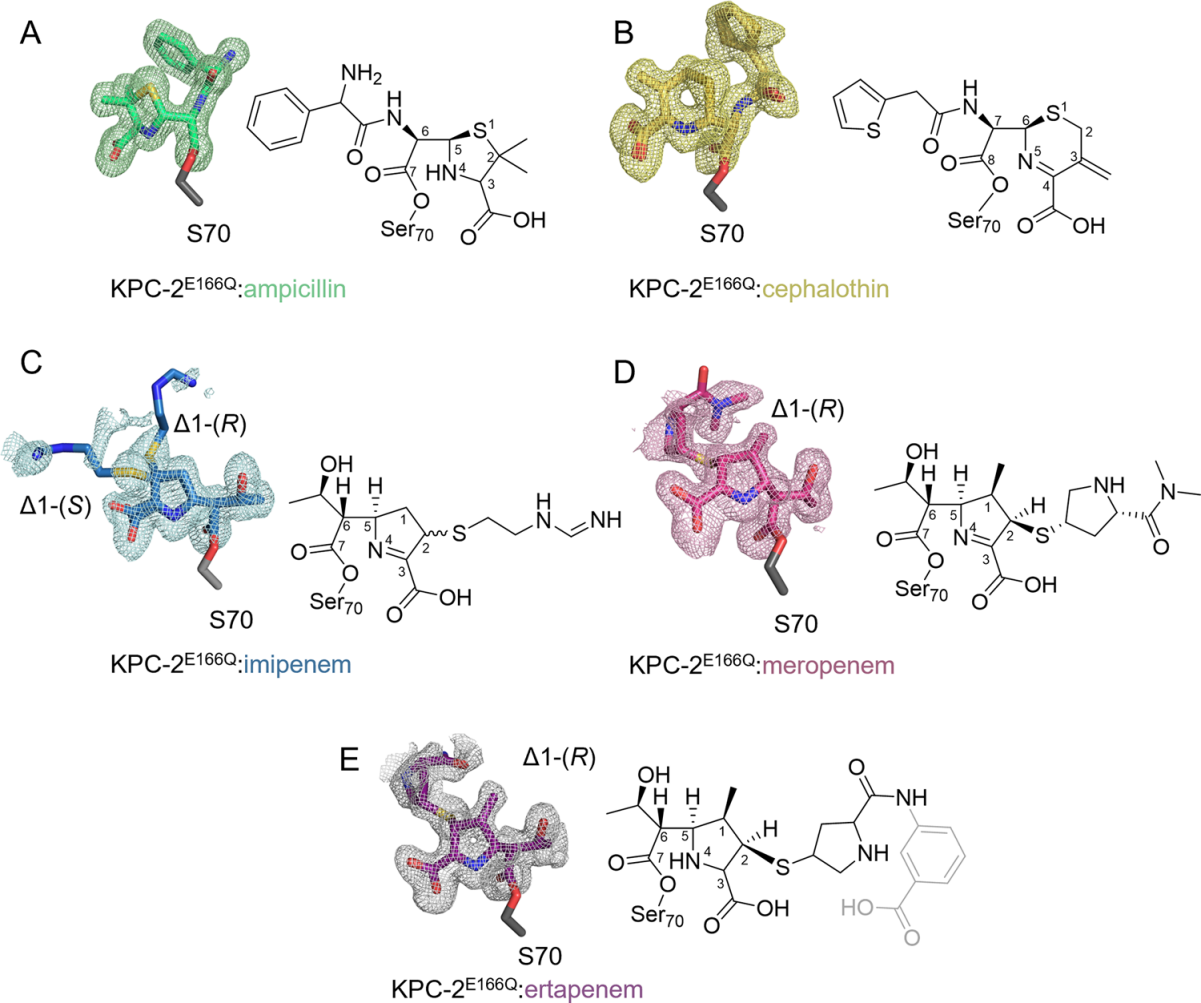
Acyl-enzyme complexes of KPC-2^E166Q^. *F*_o_ – *F*_c_ electron-density maps (colored mesh, contoured at 3σ) are calculated from the final model with the ligand omitted. (A) Ampicillin-derived acyl-enzyme (green; PDB: 8AKI). (B) Cefalothin-derived acyl-enzyme (yellow; PDB: 8AKJ). (C) Imipenem-derived acyl-enzyme (blue; PDB: 8AKK). Note: the dual conformation of the ligand (C-2 atom in both *R* and *S* configurations) is clearly defined by electron density of the R-group sulfur. (D) Meropenem-derived acyl-enzyme (pink; PDB: 8AKL). (E) Ertapenem-derived acyl-enzyme (purple; PDB: 8AKM); atoms in gray were not modeled due to the poorly defined electron density in this region.

To investigate the interactions of members of these different antibiotic classes with the KPC-2 active site, we utilized the isosteric Glu166Gln substitution (KPC-2^E166Q^)^13^ to slow the deacylation of the acyl-enzyme (allowing resolution of the acyl-enzyme intermediate). Pre-formed KPC-2^E166Q^ crystals were soaked with solutions of ampicillin, cefalothin, imipenem, meropenem, or ertapenem, representing three different β-lactam antibiotic classes (penicillins, cephalosporins, and carbapenems) for a range of exposure times, and diffraction data were collected. The resulting data extended to a high resolution (1.25–1.4 Å) (Table S1) with a clear *F*_o_ – *F*_c_ difference density in the KPC-2 active sites, enabling confident modeling of the antibiotic-derived complexes in which the cleaved antibiotic is covalently bonded to Ser70 (Figure 4). Real-space correlation coefficient (RSCC) values of 0.89–0.97 (as calculated by the wwPDB validation server;^57^Table S2) show good fit of the modeled ligands to the experimental electron-density maps. As observed in other crystal structures of SBL:cephalosporin acyl-enzyme complexes,^13^ the 3′ leaving group of the cefalothin-derived product could not be modeled and has most likely been eliminated during rearrangement of the acyl-enzyme. The cefalothin C-7 thiophenylacetamido substituent was modeled in two ring-flipped conformations (occupancies of 0.91/0.09) that differ in the orientation of the thiophenyl ring (Figure S3). The penicillin C-6 2-amino-2-phenylacetamido substituent was modeled as a single conformation in which all atoms could be resolved. By contrast, the C-2 substituents of the three carbapenem-derived acyl-enzymes are less well defined by the experimental electron density. In particular, the benzoic acid-derived atoms of the ertapenem side chain could not be modeled, indicating the considerable flexibility of the C-2 groups of carbapenem-derived acyl-enzymes within the KPC-2 active site (Figure S4). For all the structures presented here, KPC-2 could be modeled as a continuous chain (residues 25–293) with the respective Ω-loops (containing Gln166) all present in single conformations. This contrasts with our previously published structure of the KPC-2^E166Q^:faropenem-derived acyl-enzyme complex in which two conformations of Gln166 were observed, oriented either “in” or “out” of the active site.^56^

The acyl-enzymes (PDBs 8AKI–8AKM; Table S1) show H-bonds with the side-chain hydroxyl of Ser130, which acts as a H-bond acceptor to protonated N-4 of bound ampicillin or as a H-bond donor to the imine (unprotonated) N-4/N-5 of bound carbapenems and cephalosporins. Similar interactions were also observed in our previous structures of the KPC-2 acyl-enzymes formed on a reaction with cefotaxime, ceftazidime,^13^ and faropenem.^56^ Comparison with the uncomplexed enzyme (PDB: 5UL8)^32^ reveals that Asn132 retains the H-bond network with Lys73 and Gln166 while forming an additional H-bond with the C-6/C-7 amide carbonyl oxygen of bound penicillins/cephalosporins or the 6α-hydroxyethyl hydroxyl in acyl-enzymes of penems/carbapenems.

### KPC-2:Carbapenem Acyl-Enzymes Are Present in Crystals as Δ1 Tautomers

**Figure 5:**
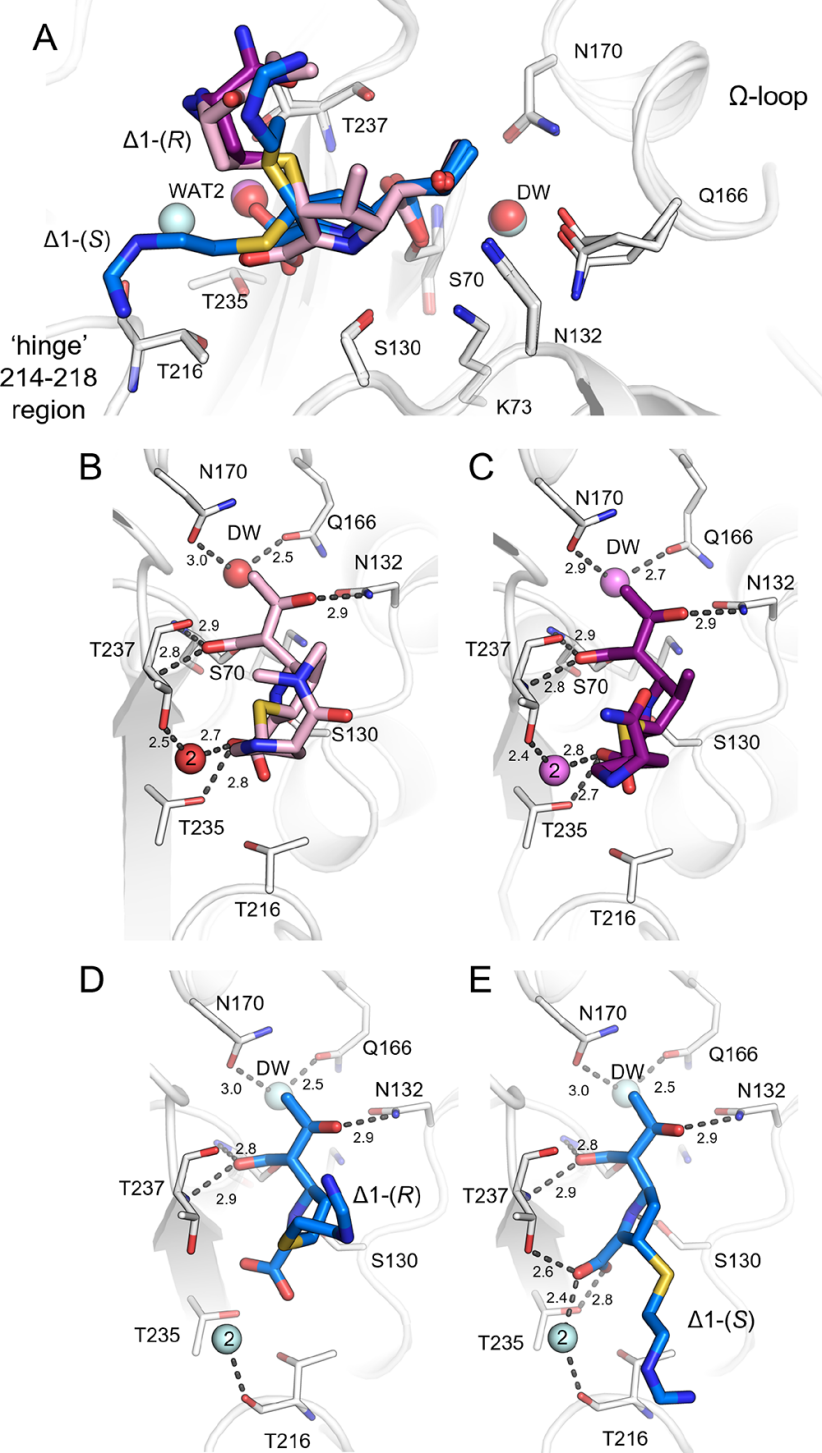
Hydrogen-bonding interactions in the KPC-2^E166Q^:carbapenem acyl-enzyme complex structures. (A) Superposition of KPC-2^E166Q^ acyl-enzyme complexes with imipenem (blue), meropenem (pink), and ertapenem (purple). (B) KPC-2^E166Q^:meropenem. (C) KPC-2^E166Q^:ertapenem acyl-enzyme complexes. (D, E) KPC-2^E166Q^:imipenem in the Δ1-(2*R*) and Δ1-(2*S*) configurations. The protein backbone is represented as a white cartoon with active-site residues as thin sticks. The deacylating water (DW) and a second active-site water interacting with the carbapenem carboxylate (**2**) are shown as colored spheres. Interactions are shown as dashed gray lines with distances (Å) labeled.

In all cases, the carbapenem-derived acyl-enzymes exist in the KPC-2 active site as Δ1-pyrroline (imine) tautomers as evidenced by the position of the exocyclic sulfur, which, at these resolutions/occupancies, can be clearly resolved as out of the plane of the pyrroline ring (Figures 4 and 5). The C-2 atom is therefore, at least predominantly, sp^3^-hybridized in all cases; in the case of imipenem, the carbapenem acyl-enzymes were modeled into the experimental electron density as 2*R* and 2*S-*enantiomers in dual occupancy (imipenem occupancy of 0.5/0.5 for the 2*R* and 2*S*-forms, respectively), while the 1β-methyl carbapenems meropenem and ertapenem were modeled as a Δ1-(2*R*)-enantiomer only. In all cases, the β-lactam-derived C-7 carbonyl of the antibiotic-derived acyl-enzyme is positioned in the oxyanion hole formed by the backbone amides of Ser70 and Thr237, and the 6α-hydroxyethyl oxygen atom is positioned to H-bond with the side-chain nitrogen of Asn132 (Figure 5 and Figures S5 and S6).

In the meropenem and ertapenem Δ1-(2*R*) acyl-enzymes, the C-3 carboxylate interacts with the side-chain oxygen atoms of Thr235 and, via a water molecule (Wat2; Figure 5 and Figures S5 and S6), Thr237. In contrast, in the Δ1-(2*S*)-imipenem acyl-enzyme, the C-3 carboxylate is rotated by 90° with respect to its position in the Δ1-(2*R*)-imipenem acyl-enzyme (Figure 5) and interacts with Thr237 and Thr235 and via a water molecule (possibly equivalent to that displaced from the Wat2 position (Figure 5 and Figures S5 and S6)) with the backbone carbonyl oxygen of Thr216 in the “hinge” region. The rotation of the C-3 carboxylate group in the Δ1-(2*S*)-imipenem acyl-enzyme is necessary to avoid a steric clash with the exocyclic sulfur of the C-2 substituent. In the Δ1-(2*R*)-imipenem acyl-enzyme, the C-3 carboxylate is in a similar position to that observed in the Δ1-(2*R*) meropenem and ertapenem acyl-enzymes, but the H-bond interactions with the Thr237 and Thr235 side chains and Wat2 are lost (Figures S5 and S6), which is probably due to the displacement of Wat2 (pale blue sphere), which is necessary to support the exchange of the Δ1-(2*R*) acyl-enzyme with its (2*S*)-stereoisomer.

### QM/MM Simulations Reveal Preferential Deacylation of the Meropenem Δ2 Tautomer

The crystallographic observation of the Δ1 form in the KPC-2 carbapenem acyl-enzymes was unexpected, given previous reports that such complexes of other class A SBLs are hydrolytically inert^12,58−60^ and our previous crystallographic observation of the meropenem acyl-enzyme of the related carbapenemase SFC-1 (trapped using a Glu166Ala mutation) in the Δ2 form.^14^ Thus, to investigate the behavior of the carbapenem-derived KPC-2 acyl-enzyme in both the Δ1 and Δ2 forms, the KPC-2^E166Q^:meropenem acyl-enzyme structure was used as a starting point for molecular dynamics (MD) simulations. In order to simulate native KPC-2, Gln166 was replaced with Glu *in silico* (Figures S7 and S8), which is a change that previously showed no significant effect upon the dynamics of the uncomplexed enzyme in triplicate 100 ns MM MD simulations.^13^ The Δ2-pyrroline (enamine) meropenem-derived acyl-enzyme was modeled using the X-ray structure of the Δ1-(2*R*)-pyrroline (imine) as a guide. In total, four systems (KPC-2^E166Q^:meropenem-Δ2, KPC-2:meropenem-Δ2, KPC-2^E166Q^:meropenem-Δ1-(2*R*), and KPC-2:meropenem-Δ1-(2*R*)) were evaluated in 500 ns triplicate MM MD simulations (for a total of 1.5 μs of MD for each system). (The Δ1-(2*S*) form was not simulated because it was not observed in either the meropenem or ertapenem acyl-enzyme complexes. This is consistent with solution NMR studies of KPC-2-catalyzed carbapenem hydrolysis that identified the Δ1-(2*R*) enantiomer as formed preferentially.^61^)

No substantial differences between the different acyl-enzymes in overall root mean square deviation (RMSD) or root mean-square fluctuation (RMSF) values were observed for the duration of the simulations; and interactions of the acyl-enzyme C-7 carbonyl with the oxyanion hole and between Glu166 and Asn170 were similarly maintained, as described in Figures S7–S9 and Note S1. Specifically, during triplicate 500 ns simulations of the KPC-2:meropenem acyl-enzymes, the acyl-enzyme carbonyl oxygen remained within the oxyanion hole with the exception of 1 ns (for each individual repeat) of simulations of the Δ2 tautomer. The observation that the KPC-2:meropenem acyl-enzyme carbonyl remains within the oxyanion hole of the KPC-2 active site is consistent with the carbapenemase activity of KPC-2 (Figure S9) and contrasts with observations of carbapenem acyl-enzymes of other class A enzymes that lack efficient carbapenem-hydrolyzing activity.^9,59,62^ The 6α-hydroxyethyl group adopts very similar positions in the (energy-minimized and equilibrated) starting structures for simulations of the KPC:meropenem acyl-enzymes in both the Δ1-(2*R*) and Δ2 configurations (hydrogen-bonded to Asn132, dihedral, c. −160°; Figure S10); however, in both the MM and QM/MM (see below) simulations, the 6α-hydroxyethyl group samples a wider range of dihedral angles in the Δ2 acyl-enzyme, accessing an orientation with a dihedral of c. −70° that is not populated in simulations of the Δ1-(2*R*) tautomer (Figure S10). Importantly, orientations (dihedral, c. 50°) involving hydrogen bonds to the deacylating water molecule (DW) are only sparingly sampled in the MD simulations (occurring in only 4.7 and 1.3% of frames in simulations of the Δ2 and Δ1-(2*R*) meropenem tautomers, respectively).

To investigate the stability of the two acyl-enzyme tautomers, we employed DFT calculations to determine the relative energies of the Δ2 and Δ1-(2*R*) acyl-enzyme complexes in two models: a small model of the acyl enzyme (containing only the side chain of Ser70 bound to the antibiotic) in implicit aqueous solvent without active site residues and a larger model including active site residues (Figure S11). In both models, the Δ1-(2*R*) tautomer is the lower-energy state (i.e., more stable than the Δ2 tautomer) with relative energies (Δ*E*_Δ2−Δ1(2*R*))_) of +5.7 and +19.9 kcal/mol in the small model and active site model, respectively. These results are consistent with our crystallographic observation of the meropenem Δ1-(2*R*) acyl-enzyme as well as with solution studies of carbapenem hydrolysis products.^61^

QM/MM simulations, starting from the KPC-2 meropenem acyl-enzyme structures prepared for the molecular dynamics simulations above, were then employed to investigate the energetics of deacylation of the KPC-2:meropenem acyl-enzymes in both the Δ1-(2*R*) and Δ2-pyrroline forms. As in our previous work,^15,21^ we focused on the first stage of deacylation, namely, the attack of water on the acyl-enzyme carbonyl carbon to form a tetrahedral deacylation intermediate (TI) via a transition state (TS). Henceforth, we use the term TS when referring to the transition state for TI formation from the acyl-enzyme complex. This first step was simulated because it represents the probable rate-limiting step for the deacylation reaction. Our previous QM/MM simulations of this step accurately differentiated between class A enzymes with carbapenemase activity and others that are inhibited by carbapenems.^21^

TI structures were obtained from conventional umbrella sampling simulations of the 2D MFEP for meropenem deacylation (QM region defined in Figure S2) with reaction coordinates defined as described above and in Note S1. The recently introduced adaptive string method (ASM)^23,63^ finds the minimum free energy pathway (MFEP) (“on the fly”) for reactions in the space of a set of collective variables, sampling to obtain a free energy profile of the process. Both conventional umbrella sampling and the ASM QM/MM simulations were used to calculate free energy profiles for the first stage of deacylation of the meropenem acyl-enzymes in the Δ2 and Δ1-(2*R*) configurations. These ASM calculations identified a clear difference between the two tautomers with respect to the energetics of tetrahedral intermediate formation with the Δ2 tautomer undergoing this reaction much more readily (Δ*G*^⧺^ = 12.3 ± 3.5 kcal/mol) than the Δ1-(2*R*) tautomer (Δ*G*^⧺^ = 19.4 ± 1.6 kcal/mol) (Figure S12 and Table S3). While umbrella sampling gave similar results (Table S3), the ASM method is less biased and gives a path closer to the true MFEP, more clearly differentiating between the two carbapenem tautomers and their MFEPs, so we focus on these results.

### Arrangement and Stability of H-Bond Networks in the Deacylation Transition State

**Figure 6:**
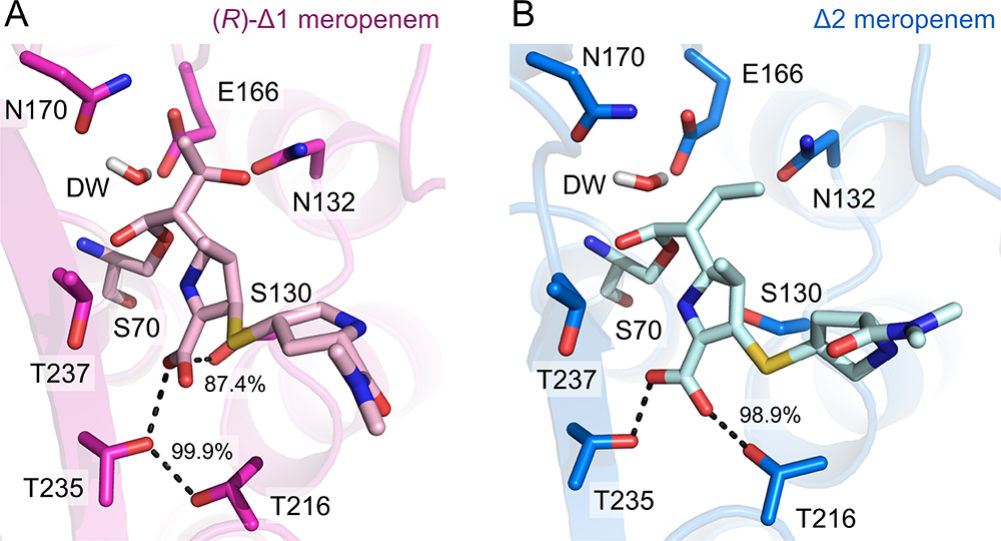
ASM calculations of meropenem tetrahedral intermediate formation reveal changes in hydrogen-bonding networks around the C-3 carboxylate of KPC-2:meropenem transition states. Snapshots taken of (A) the KPC-2:Δ1-(*2R*)-meropenem and the (B) KPC-2:Δ2-meropenem complexes showing the proximity of the deacylating water (DW) proton to E166 (proton transfer) and the DW oxygen to the meropenem C-7 carbonyl carbon (nucleophilic attack) in the transition state (TS) before the formation of the tetrahedral intermediate (TI). Key differences in the hydrogen-bond networks between the protein main chain and the meropenem C-3 carboxylate are shown as dashes. These are labeled with the corresponding percentage of H-bonds formed over the trajectory.

To investigate the basis for the differences in barrier heights of TI formation between the Δ1-(2*R*) and Δ2 pyrroline forms of the KPC-2:meropenem acyl-enzyme, the respective TSs for TI formation obtained from ASM QM/MM simulations were compared (Figure 6 and Figure S13 and Table S4). This revealed differences between the Δ1-(2*R*) and Δ2 pyrroline forms in the H-bonding networks in the KPC-2 active site (Figure 6 and Figure S13 and Table S4). For this analysis, H-bonds were defined by the distance and angle (calculated by CPPTRAJ)^35^ between the protein and meropenem for structures from MFEP simulations.

Both meropenem tautomers make common H-bonds to KPC-2, specifically between the acyl-enzyme carbonyl oxygen (MER O27) and the backbone amides of Ser70 and Thr237 of the oxyanion hole and between Ser70 Oγ and the side-chain Nζ of Lys73. Additional H-bonds are, however, tautomer-specific (Figure 6 and Figure S13 and Table S4); the meropenem C-3 carboxylate predominantly makes H-bonds to the side chains of Thr216 (98.9% of frames) and Thr235 (99.6%) in the Δ2 tautomer, but to those of Thr235 (99.9%) and Ser130 (87.4%) in the Δ1-(2*R*)-derived structure.

During the simulated reaction, the Δ1-(2*R*) and Δ2 tautomers also differ with respect to their H-bonding patterns involving the DW (Table S6). For the Δ1-(2*R*)-meropenem acyl-enzyme, H-bonds to the DW were promiscuous and involved either the Oδ1 (64.9% of all ASM frames) or Nδ2 (6.1%) atoms of Asn170 and the Oε1 (22.6%) or Oε2 (51.5%) atoms of Glu166 (Table S6). In contrast, the KPC-2:Δ2-meropenem complex had more consistent H-bond donors/acceptors with the DW in the simulations; H-bonds to Asn170 occurred most often with Oδ1 (74.8% of frames and only 0.3% with Nδ2) and to Glu166 with Oε1 (75.4% and 5.8% with Oε2) (Table S6). Compared to the Δ1-(2*R*) configuration, H-bonds to the DW were not only more consistent in QM/MM simulations of the KPC-2:Δ2-meropenem species but were also 10% more frequent (Table S6). In simulations with meropenem in the Δ1-(2*R*) configuration, Glu166 and Asn170 and the DW sampled more conformational space and participated in fewer interactions than the Δ2 tautomer. There were also small differences in the DW to the C-7 angle (i.e., the angle of nucleophilic attack for deacylation) in which the Δ2 tautomer sampled the favored Bürgi–Dunitz angle^64^ (i.e., between 107 and 109°) in 3.4% more frames than the Δ1-(2*R*) (Figure S13). In the Δ2-enamine acyl-enzyme, the DW therefore adopts a more favored orientation for proton transfer to Glu166 and a subsequent reaction with the C-7 carbonyl carbon. In contrast, in the Δ1-(2*R*)-imine configuration, the DW is less well-positioned, spending more time in positions that are disfavored for either nucleophilic attack or proton transfer events.

## Discussion

**Figure 7:**
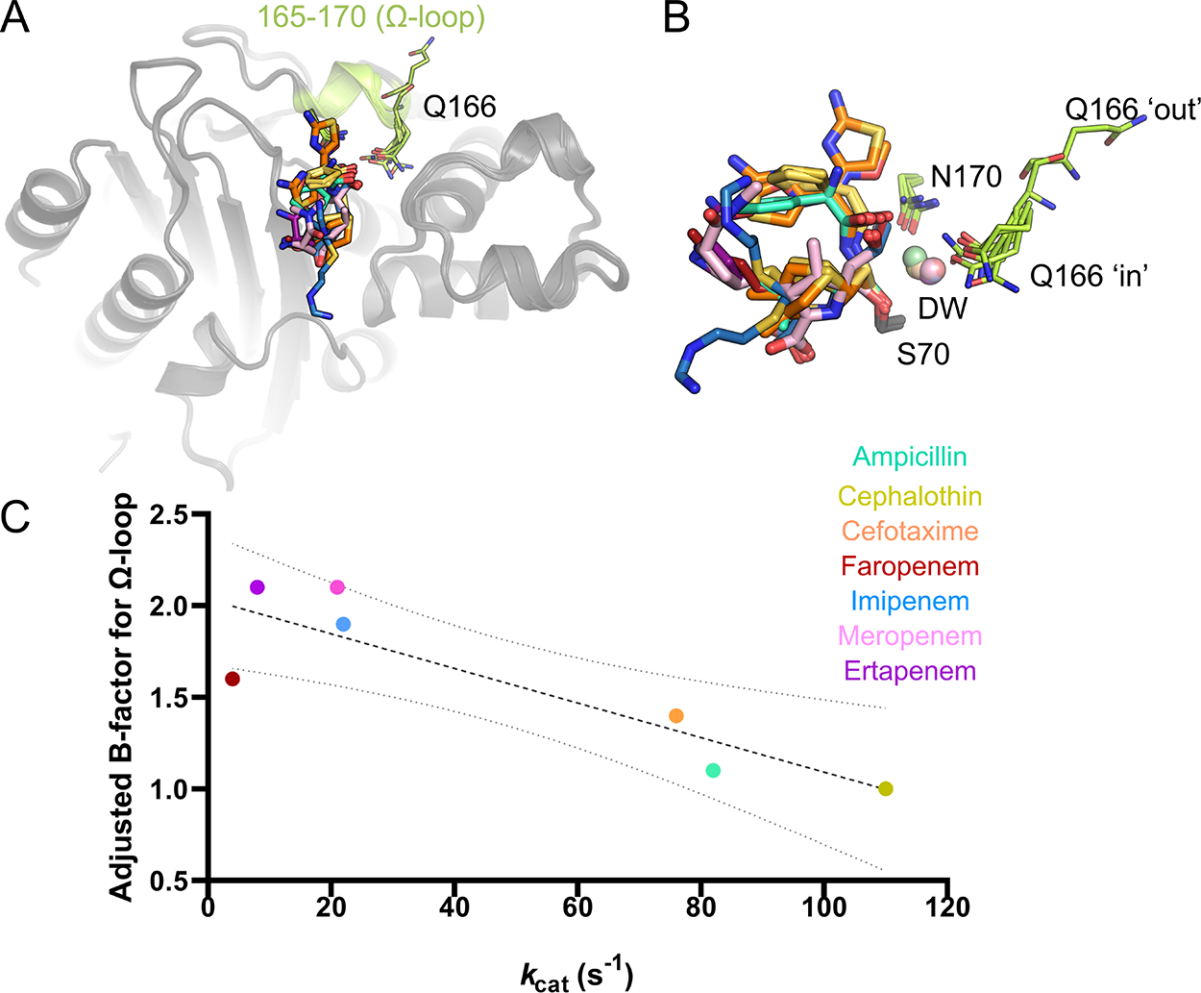
B-factors of the KPC-2 Ω-loop for β-lactam acyl-enzyme complex structures correlating with *k*_cat_. (A) Overall view of KPC-2 active site with antibiotic acyl-enzymes: residues 165–170 of the Ω-loop (highlighted in lime). (B) Zoom-in on active site positions 170 and 166; the position of the water in the putative deacylating position (DW) and corresponding antibiotic acyl-enzymes. Antibiotic-derived acyl-enzymes and DW colored as in A. (C) Values for the adjusted Ω-loop B-factor (all atoms and all conformations) and *k*_cat_ from Table S5, plotted and fit to a linear trendline with an *R*^2^ of 0.77. Dotted lines represent 95% confidence intervals for the linear regression fit. Adjusted B-factors were calculated as a ratio vs the average protein B-factor (Å^2^) within the crystal structure; see Table S5.

KPC-2 efficiently hydrolyzes a wide range of β-lactam classes, including carbapenems with varying C-2 substituents (Figure 2), and is of increasing global importance as a cause of antibiotic resistance. Our prior studies of penem (faropenem)^56^ and cephalosporin (ceftazidime and cefotaxime)^13^ turnover by KPC-2 revealed that accommodation of diverse substrates is facilitated by the mobility of loops around the active site. However, the stability of the Ω-loop and, in consequence, correct positioning of Glu166 are critical for efficient catalysis. In particular, ceftazidime binds to KPC-2 with disruption of the Ω-loop conformation, with MD simulations indicating the possibility of large movements in this region in the ceftazidime acyl-enzyme, and the potential for alternative conformations of Glu166 that position its side chain either “in” to or “out” of the active site. A dual in/out conformation of Glu166 was also observed in a crystal structure of the faropenem-derived KPC-2 acyl-enzyme.^56^ In the “in” conformation, Glu166 would be correctly positioned to activate the water molecule for deacylation, whereas the “out” conformation does not promote hydrolysis. These findings explain the relatively poor turnover rates (*k*_cat_) shown by KPC-2 towards ceftazidime and faropenem (Table 1). In the carbapenem complexes described here, disruption of the Ω-loop is less apparent with, in each case, the loop being modeled in a single conformation in which all residues are resolved. However, analysis of the B-factors of the Ω-loop (residues 165 to 170) in structures of KPC-2^E166Q^ acyl-enzyme complexes other than ceftazidime (for which a weak electron density precluded modeling of the Ω-loop,^13^ that is, with ampicillin, cefalothin, cefotaxime, imipenem, meropenem, ertapenem, and faropenem) shows a negative correlation with steady-state *k*_cat_ values (Figure 7 and Table 1), indicating that faster antibiotic turnover rates are related to a lower average mobility of the Ω-loop. In particular, the stability and rigidity of the conformations of Glu166 and Asn170 are crucial for productive interactions with the deacylating water molecule^5,13,56^ and for keeping conserved, catalytically important, active-site H-bonding networks intact. In the case of carbapenems, therefore, efficient deacylation can in part be attributed to the relatively well-defined conformations of the Ω-loop in the relevant acyl-enzyme complexes, although comparison with substrates of other classes (e.g., ampicillin and cephalothin) for which the turnover is faster and Ω-loop B-factors are lower indicates that these are not completely optimal.

X-ray crystal structures are now available for carbapenem acyl-enzymes of multiple class A SBLs (Figure S14). These enable the comparison of carbapenem binding across several class A β-lactamases including carbapenemases (SFC-1,^14^ GES-5,^59^ and KPC-2) and carbapenem-inhibited enzymes (TEM-1,^58^ SHV-1,^22^ GES-1,^59^ and BlaC^65^). Several features of carbapenem binding have been proposed to be important to efficient turnover,^5^ including the positioning of the C-7 carbonyl group within the oxyanion hole. In the TEM-1 and SHV-1 carbapenem acyl-enzymes, there is evidence that the acyl-enzyme carbonyl group can “flip” out of this site, retarding deacylation.^22,58^ In contrast, in the structures that we report here, the acyl-enzyme carbonyl is invariably positioned in the oxyanion hole and only very transiently moves outside it in the molecular simulations.

Efficient carbapenem deacylation has also been associated with orientations of the 6α-hydroxyethyl substituent that prevent interactions with the deacylating water molecule (DW) that may reduce its nucleophilicity.^14^ The structures and simulations presented here show that, in KPC:meropenem complexes, this group is consistently positioned to interact with Asn132 and beyond the H-bonding distance of the DW. Although both MD and ASM simulations identify that conformations (dihedral +50°) bringing the 6α-hydroxyethyl closer to the DW are occasionally sampled by the meropenem acyl-enzyme (Figure S10), the low frequencies with which these occur support the conclusion that preventing interactions of the 6α-hydroxyethyl group with the DW is important to the carbapenem-hydrolyzing ability of class A enzymes such as KPC-2.

The efficiency of carbapenem hydrolysis by SBLs is thought to be affected by the ability of the carbapenem-derived products to tautomerize between the Δ1-(2*R*/2*S*) and Δ2 forms (Figure 3). Our previous NMR studies indicated the formation of products as the Δ2 tautomer by multiple carbapenemases followed by rapid tautomerization to the Δ1-(2*R*) form.^61^ Similarly, kinetic^66,67^ and Raman^12^ studies on carbapenem-inhibited enzymes (i.e., TEM-1 and SHV-1) indicated an initial formation of the acyl-enzyme in the Δ2 tautomer, which subsequently either deacylates or isomerizes to the Δ1 form(s). The present crystallographic data, which identify acyl-enzymes of KPC-2 with multiple carbapenems as all existing in the Δ1-(2*R*) form, provides evidence that this isomerization can also take place in carbapenem-hydrolyzing enzymes. Specifically, our high-resolution crystal structure of the KPC-2:imipenem-derived acyl-enzyme reveals that both the (2*R*) and (2*S*) stereoisomers of the Δ1 tautomer coexist, suggesting that, at least in this system, tautomer interconversion has a low energy barrier and can occur on (crystallized) KPC-2 after an initial formation of the Δ2 acyl-enzyme (Figure 3). In previous investigations of carbapenem-inhibited enzymes, accumulation of the Δ1 acyl-enzyme led to its designation as (near-)hydrolytically inert. Consistent with these observations, using the ASM, our QM/MM simulations of the formation of the deacylation TI show a significantly higher barrier for deacylation of the Δ1-(2*R*) tautomer of meropenem than for the Δ2 form (19.4 ± 1.6 kcal mol^–1^ compared to 12.3 ± 3.5 kcal mol^–1^; Table S3).

The likely basis for differences in the susceptibility to hydrolysis of the Δ1-(2*R*) and Δ2 tautomers is explained by analysis of the ASM simulations. Previous studies indicate that the 6α-hydroxyethyl substituents of carbapenem acyl-enzymes can adopt multiple positions and orientations in different class A β-lactamases.^5,14,58,62^ This enables interactions with either Glu166 (as seen in the BlaC:doripenem acyl-enzyme, PDB 3IQA; Figure S14^58^) or the DW (SHV-1:meropenem acyl-enzyme, PDB 2ZD8, Figure S14^62^). Our previous MD study of the meropenem acyl-enzyme of the efficient carbapenemase SFC-1 showed that the meropenem 6α-hydroxyethyl group made persistent H-bonds with Asn132,^14^ preventing contacts with the DW that would slow deacylation, explaining the basis for SFC-1 carbapenemase activity.^14^ The simulations reported here are consistent with these findings (see above). However, our QM/MM simulations reveal further contributors to efficient carbapenem hydrolysis by KPC-2 through comparisons of the TSs of the two meropenem-derived tautomers.

Specifically, hydrogen bonding around the meropenem C-3 carboxylate (Figure 6) requires mutually exclusive networks in the Δ1-(2*R*) and Δ2 tautomer complexes that involve residues Thr216, Thr235, and Ser130 and result in the C-3 carboxylate adopting different orientations in the two tautomers. In consequence, the N-4 N–H group of the Δ2 (enamine) tautomer will be electrostatically stabilized by proximity to the O-9 oxygen. Furthermore, the proximity of the enamine N-4 N–H to the acyl-enzyme ether Oγ and carbonyl oxygen atoms provides for further electrostatic interactions, which will strengthen during the formation of the deacylation TS (as the nascent oxyanion accumulates negative charge) and TI (**5**, Figure 1). This will stabilize these species relative to the starting acyl-enzyme with consequent reduction in the free-energy barrier for deacylation. In contrast, the unprotonated imine N-4 of the Δ1-(2*R*) tautomer can experience no such stabilizing effect, and indeed, the proximity of its lone pair to Ser70 Oγ is expected to be relatively destabilizing for a tetrahedral intermediate formation.

Furthermore, the two tautomers differ in the interaction networks involving the deacylating water molecule (DW). In simulations of the Δ2 tautomer, the DW makes consistent H-bonds to specific atoms in the Asn170 and Glu166 side chains, while for the Δ1-(2*R*) tautomer, interactions of DW with its H-bonding partners are more variable. Therefore, in the Δ2-enamine form of the KPC-2:meropenem acyl-enzyme, the position and orientation of the DW in the TS are more constrained, facilitating its addition to the acyl-enzyme carbonyl carbon. Taken together with the stabilizing contributions of charge-based interactions involving protonated N-4 in the deacylation transition state and intermediate, this results in a lower free energy barrier for TI formation and hence a more rapid deacylation.

Alongside these effects upon the TS and TI species derived from the different meropenem tautomers, our DFT calculations also identify differences in stability between the meropenem Δ2 and Δ1-(2*R*) acyl-enzyme complexes. Specifically, these show the Δ1-(2*R*) acyl-enzyme tautomer to be intrinsically more stable than the Δ2 both in a small model containing the covalently bound ligand and Ser70 only and in a more extensive model that includes relevant active-site residues. Thus, the barrier to deacylation of the meropenem Δ2 acyl-enzyme is reduced both by stabilization of the transition state and destabilization of the starting acyl-enzyme relative to the equivalent species for the Δ1-(2*R*) tautomer.

Our crystallographic observation of KPC-2:carbapenem acyl-enzymes in the Δ1 form (obtained using deacylation-deficient mutants) might be considered surprising as it could be expected that their formation by rearrangement of the Δ2 species might be disfavored or prevented in carbapenem-hydrolyzing class A enzymes such as KPC-2. Another possibility is that class A carbapenemases might instead accelerate the deacylation of Δ1 acyl-enzymes such that these can no longer accumulate as inhibitory species. Our data suggest however that neither of these are the case, that is, the carbapenem-hydrolyzing activity of KPC-2 and by implication other class A enzymes with carbapenemase activity arises primarily from a fast rate of deacylation of the Δ2 acyl-enzyme as evidenced by the low barriers obtained from QM/MM simulations. A further question is then how KPC-2 and related enzymes avoid the accumulation of the Δ1 acyl-enzyme in solution that our data identify as resistant to deacylation. In this respect, our crystallographic observation of the imipenem acyl-enzyme in both Δ1-(2*R*) and Δ1-(2*S*) configurations is significant as it suggests that these species are in exchange in the acyl-enzyme form, that is, that tautomerization via the Δ2 form is reversible. Thus, efficient carbapenem turnover is possible as interconversion between tautomers ensures that the acyl-enzyme retains access to a state (Δ2) that is deacylation-competent even though the Δ1 forms remain poorly hydrolyzed. The greater stability of the Δ1-tautomers compared to the Δ2-form,^61,68^ as also evidenced here in both DFT and ASM calculations, would then explain the accumulation of the former *in crystallo* in the deacylation-deficient mutant employed here.

Identification of the structural features of KPC-2 and related class A β-lactamases that are responsible for their efficient carbapenem-hydrolyzing activity is a long-standing challenge in the field. The results described here, taken together with our^13,15,21^ and others’^54,69^ previous data, clarify some of the ways the KPC-2 active site is optimized to hydrolyze carbapenems in addition to other β-lactam substrates. Compared to other class A β-lactamases, KPC-2 and other carbapenemases have shallower,^70^ more open active-site clefts that enable the carbapenem 6α-hydroxyethyl group to access orientations that prevent its interaction with the DW. The presence of the Cys69–Cys238 disulfide bridge^16^ and the precise positioning of the side chains of Asn132^15,70^ and Asn/Ser170^15,71^ are also known to be important to carbapenem hydrolysis. The data presented here, however, identify additional contributors to carbapenem turnover by KPC-2. Precise positioning of the Ω-loop is clearly important to carbapenem hydrolysis as evidenced by a comparison of the structures of acyl-enzymes of multiple β-lactams (Figure 7). This conclusion is further supported by a comparison of the MD simulations of meropenem acyl-enzymes presented here, where the Ω-loop remains relatively stable, with our previous study of the poor substrate ceftazidime in which the Ω-loop is far more mobile, enabling Glu166 to access orientations that are unproductive for deacylation.

As described above, we propose that carbapenem hydrolysis by KPC-2 is enabled by a facile exchange between the different acyl-enzyme tautomers, ensuring that the deacylation-competent Δ2 configuration remains accessible. Our structural data and associated simulations identify that the interactions of the meropenem C-3 carboxylate with the KPC-2 active site differ between the two acyl-enzyme tautomers. Based on these observations, we then further hypothesize that the ability of the KPC-2 active site to accommodate alternative binding modes for the C-3 carboxylate will help support exchange between carbapenem tautomers. In this context, residues Thr216, Thr235, and Thr237, all of which interact with the C-3 carboxylate in at least one of the binding modes we describe, would be expected to be important to the carbapenamase activity of KPC-2. This proposal is consistent with the recent work of Furey *et al*.,^69^ who identified that a KPC-2 mutation of Thr216 or Thr237 affects carbapenem turnover over that of other β-lactams, and their conclusion that the precise conformation of the loop formed by residues 214–220 is important in KPC-2 carbapenemase activity. The acyl-enzyme binding modes that we observe are consistent with those previously reported for the imipenem acyl enzyme (in the Δ1-(2*S*) configuration) bound to the KPC-2 Phe72Tyr mutant and with non-covalent complexes of hydrolyzed imipenem bound to the Asn170Ala (Figure S15) and Ser70Gly/Thr215Pro mutants.^69^ In comparison, it is notable that hydrolyzed ampicillin adopts a different binding mode to KPC-2 Asn170Ala (Figure S15).

## Conclusions

Taken together, the extensive structural data and molecular simulations presented here provide the most detailed picture to date of carbapenem hydrolysis by KPC-2. Our data show that active-site architecture, particularly that of the Ω-loop, is a crucial determinant of the rate of β-lactam antibiotic hydrolysis by this versatile enzyme. Although plasticity of the Ω-loop that enables productive binding of multiple β-lactams (penicillins, cephalosporins, and carbapenems) may be required for the broad substrate selectivity of KPC-2, increased mobility (as evidenced by crystallographic B-factor) negatively impacts turnover rate. This highlights the importance of precise positioning of Glu166 for efficient β-lactam hydrolysis. In the case of carbapenems, however, our simulations identify additional contributors to efficient turnover by KPC-2. QM/MM simulations using the ASM based upon the crystal structures reported here reveal that the meropenem acyl-enzyme in the Δ2 tautomer is much more easily deacylated than the Δ1-(2*R*) form, which is a finding that is consistent with solution analyses of products of β-lactam hydrolysis by both SBLs and MBLs. Exclusive electrostatic interactions with KPC-2 that differ between tautomers, particularly those involving the C-3 carboxylate, and the less stable interactions made by the DW in the meropenem Δ1-(2*R*) acyl-enzyme compared to the Δ2 tautomer both contribute to a 7.1 kcal/mol-higher free energy barrier for deacylation. QM/MM ASM simulations therefore have the sensitivity to identify the differing sets of interactions in the deacylation-competent (Δ2) and deacylation-incompetent (Δ1-(2*R*)) forms of the KPC-2:meropenem acyl-enzyme, which respectively promote or inhibit carbapenem turnover, and so discriminate between closely related complexes that differ in reactivity. The results thus demonstrate the utility of the ASM method in QM/MM simulations as a tool with which to investigate the energetics of enzyme-catalyzed reactions (and effects of substrate modifications and enzyme mutations). These findings also suggest that modifying β-lactams to promote the formation of Δ1-type acyl-enzyme complexes represents one possible route to overcoming β-lactam resistance caused by class A carbapenemases.

## Data Availability

For all crystal structures presented herein, atomic coordinates and structure factors have been deposited to the Worldwide Protein Data Bank (PDB; wwpdb.org) under accession codes 8AKI (KPC-2^E166Q^:ampicillin), 8AKJ (KPC-2^E166Q^:cephalothin), 8AKK (KPC-2^E166Q^:imipenem), 8AKL (KPC-2^E166Q^:meropenem), and 8AKM (KPC-2^E166Q^:ertapenem). Example structures of the acyl-enzyme complex, transition state, and tetrahedral intermediate of both tautomers and ligand parameter files for all simulations are available at the University of Bristol Research Data Repository (https://data.bris.ac.uk/data).

## Supporting Information Available

Crystallographic data collection and refinement, analyses of simulation data, extended analyses of crystallographic and simulation data, and additional details of methods and analysis for MD simulations and QM/MM calculations (PDF)
