# Supplementary material for: Tautomer-Specific Deacylation and Ω-Loop Flexibility Explain the Carbapenem-Hydrolyzing Broad-Spectrum Activity of the KPC-2 β-Lactamase
Source: J Am Chem Soc. 2023 Mar 27;145(13):7166–80. doi: 10.1021/jacs.2c12123 (PMC10080687; doi:10.1021/jacs.2c12123)
Supplement: Supplementary file 1 — ja2c12123_si_001.pdf [file ja2c12123_si_001.pdf]

Supporting information for

Tautomer-specific deacylation and Ω-loop flexibility explain carbapenem-hydrolyzing, broad-spectrum activity of the KPC-2 β-lactamase

Catherine L. Tooke[1], Philip Hinchliffe[1]◊, Michael Beer[1, 2]◊, Kirill Zinovjev[3,4], Charlotte K. Colenso[1, 2], Christopher J. Schofield[5], Adrian J. Mulholland[2] and James Spencer[1].*

1. School of Cellular and Molecular Medicine, Biomedical Sciences Building, University Walk, University of Bristol, Bristol, BS8 1TD, United Kingdom
2. Centre for Computational Chemistry, School of Chemistry, Cantock's Close, University of Bristol, Bristol, BS8 1TS, United Kingdom
3. School of Biochemistry, Biomedical Sciences Building, University Walk, University of Bristol, Bristol, BS8 1TD, United Kingdom
4. Departamento de Química Física, Universitat de València, Burjassot, 46100, Comunitat Valenciana, Spain
5. Chemistry Research Laboratory, Department of Chemistry and the Ineos Oxford Institute for Antimicrobial Research, Mansfield Road, University of Oxford, Oxford, OX1 3TA United Kingdom.

◊ P.H. and M.B. contributed equally to this work.

* Corresponding author Email: jim.spencer@bristol.ac.uk

Contents

Tables

Table S1. Data collection and refinement statistics for KPC-2^{E166Q} crystal structures.

Table S2. Ligand statistics from KPC-2 crystal structures presented herein

Table S3. Calculated activation free energy barriers for meropenem derived complex deacylation by KPC-2.

Table S4. H-bonds between the meropenem (MER) acyl-enzyme complex and the KPC-2 protein mainchain observed during transition state frames in QM/MM calculations.

Table S5. Crystallographic B-factor comparisons with k_{cat} values

Table S6. Hydrogen bond interactions observed for of the deacylating water over adaptive string method (ASM) simulations

Table S7. Active site distances in molecular dynamics (MD), umbrella sampling (US) and adaptive string method (ASM) calculations

Table S8 Solvent accessible surface area (SASA) in Å^2 for the KPC-2 active site during KPC-2:meropenem simulations

Figures

Figure S1. View of the KPC-2 apo-enzyme active site.

Figure S2. QM region (black) used for QM/MM calculations.

Figure S3. Evidence for two conformations of the thiophenylacetamido C-7 substituent in the KPC-2^{E166Q}:cefalothin complex.

Figure S4. Carbapenem-derived acyl-enzyme complexes colored with respect to atomic B-factor.

Figure S5. Possible hydrogen bonding interactions in KPC-2^{E166Q} carbapenem acyl-enzyme complexes.

Figure S6. Possible hydrogen bonding interactions in KPC-2^{E166Q} carbapenem acyl-enzyme complexes.

Figure S7. RMSD plots for the KPC-2:meropenem complex over triplicate 500ns simulations.

Figure S8. Per residue RMSF plots for the KPC-2:meropenem-derived acyl-enzyme complex over triplicate 500 ns simulations.

Figure S9. Distance analyses over KPC-2:meropenem-derived acyl-enzyme triplicate simulations.

Figure S10. Position of the hydroxyethyl in MM and QM simulations.

Figure S11. DFT calculations on meropenem acyl-enzymes.

Figure S12. ASM calculations for tetrahedral intermediate formation from the meropenem-derived acyl-enzyme in KPC-2 catalysis.

Figure S13. Transition state analysis of adaptive string method calculations.

Figure S14. Comparisons of the interactions of carbapenem acyl-enzymes within Class A SBLs.

Figure S15. Comparison of KPC-2:antibiotic acyl-enzyme and non-covalent hydrolysis product complexes.

Figure S16. Ser130 interactions throughout MD and ASM simulations.

Figure S17. Lys234 networks throughout ASM simulations.

Supporting notes:

Supporting note 1

Supporting note 2

Tables

Table S1. Data collection and refinement statistics for KPC-2^{E166Q} crystal structures

	KPC-2^{E166Q}: ampicillin	KPC-2^{E166Q}: cephalothin	KPC-2^{E166Q}: imipenem	KPC-2^{E166Q}: meropenem	KPC-2^{E166Q}: ertapenem
**PDB ID**	8AKI	8AKJ	8AKK	8AKL	8AKM
**Data collection**					
**Space group**	$P2_12_12$	$P2_12_12$	$P2_12_12$	$P2_12_12$	$P2_12_12$
**Molecules/ASU**	1	1	1	1	1
**Cell dimensions**					
**a, b, c (Å)**	60.51, 79.38, 56.21	60.51, 79.58, 55.95	60.66, 79.48, 55.94	60.56, 79.21, 56.26	60.25, 78.55, 55.59
**α, β, γ (°)**	90, 90, 90	90, 90, 90	90, 90, 90	90, 90, 90	90, 90, 90
**Resolution (Å)**	48.12-1.40 (1.42-1.40)	48.17-1.35 (1.37-1.35)	48.22-1.36 (1.38-1.36)	45.86- 1.35 (1.37-1.35)	47.80-1.25 (1.27-1.25)
**R_{pim}**	0.034 (0.665)	0.032 (0.553)	0.038 (0.987)	0.030 (0.552)	0.031 (0.656)
**$CC_{1/2}$**	0.999 (0.613)	0.999 (0.726)	0.999 (0.519)	0.999 (0.858)	0.999 (0.574)
**$I/\sigma(I)$**	13.5 (1.2)	14.4 (1.2)	10.5 (0.9)	14.7 (1.3)	12.0 (1.1)
**Completeness (%)**	100 (100)	99 (97.8)	100 (100)	100 (100)	100 (100)
**Redundancy**	13.1 (13.1)	13.3 (13.6)	13.1 (13.2)	13.1 (13.3)	12.8 (12.9)
**Refinement**					
**Resolution (Å)**	48.12-1.40	48.17-1.35	48.22-1.36	45.86-1.35	47.804-1.25
**No. reflections**	54,007	59,287	58,791	60,064	73,569
**R_{work} / R_{free}**	0.157/0.1849	0.1504/0.1776	0.1542/0.1797	0.1365/0.1662	0.1511/0.1642
**No. non-H atoms**					
**Protein**	2094	2126	2125	2059	2119
**Solvent**	320	281	301	300	293
**Ligand**	24	44	40	52	66
**B-factors (Å^2)**					
**Protein**	18	17	19	18	18
**Solvent**	39	35	40	37	38
**Ligand**	17	19	30	23	34
**R.m.s. deviations**					
**Bond lengths (Å)**	0.009	0.009	0.008	0.008	0.007
**Bond angles (°)**	1.008	1.043	0.994	0.977	1.021
**Ramachandran (%)**					
**Outliers**	0.00	0.00	0.00	0.00	0.00
**Favored**	98.87	98.87	98.50	98.13	98.50

Values in parentheses are for the outer shell.

Table S2. Ligand statistics from KPC-2 crystal structures presented herein

Ligand	B-factors (Å^2)	Occupancies	RSCC (PDB)
Ampicillin	17	1.0	0.97
Cefalothin	19	0.09/0.91	0.96/0.96
Imipenem	30	0.5/0.5	0.94/0.94
Meropenem	23	1	0.89
Ertapenem	34	1	0.96

Table S3. Calculated activation free energy barriers for meropenem derived complex deacylation by KPC-2.

	Adaptive string method		Umbrella sampling	
	**Δ1**	**Δ2**	**Δ1**	**Δ2**
$\Delta^{‡}G_{calc}$	19.44 (1.57)	12.30 (3.47)	23.08 (1.14)	19.67 (1.12)

Standard deviations are shown in parentheses

Table S4. H-bonds between the meropenem (MER) acyl-enzyme and KPC-2 during transition state frames in QM/MM calculations. Main differences between tautomers are highlighted in green.

Acceptor atom	Donor atom	Adaptive string method		Umbrella Sampling	
		**Δ1**	**Δ2**	**Δ1**	**Δ2**
**MER O9**	**Thr235 Oγ1**	73.77	99.58	98.3	100
**MER O10**	**Thr216 Oγ1**	-	98.89	-	65.8
**MER O27**	**Thr237 N**	79.04	77.54	94.2	79.2
**MER O27**	**S70 N**	93.46	84.18	100	98.3
**MER O9**	**Ser130 Oγ**	87.36	-	83.3	5.8
**MER O13**	**Asn132 Nδ2**	44.06	39.56	43.3	49.2
**Asn 132 OD1**	**MER O13**	-	9.87	-	3.33
**MERO9**	**Thr237 Oγ1**	-	10.83	-	33.3
**MER O10**	**Thr235 Oγ1**	26.14	0.35	-	-
**MER O9**	**Lys234 Nζ**	6.74	-	-	-
**MER N4**	**Ser130 Oγ**	6.88	0.1	-	-

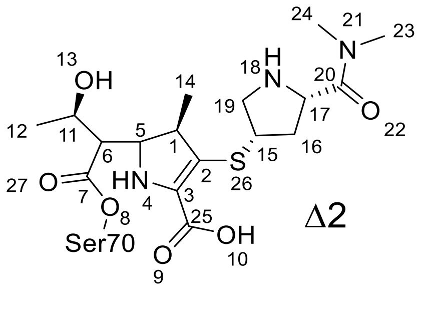

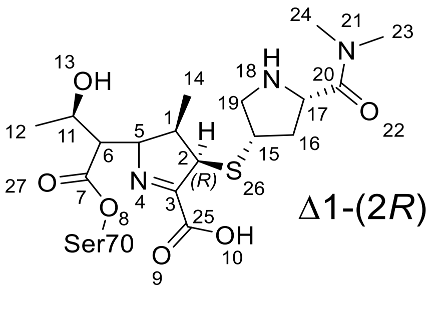

Table S5. Crystallographic B-factor comparisons with k_{cat} values

Antibiotic	k_{cat} (s^{-1})	B-factor (protein, Å^2),	B-factor (Ω-loop, Å^2)	Adjusted increase in B-factor (Ω-loop/protein)
Ampicillin	82	18	21	1.1
Cefalothin	110	17	17	1.0
Cefotaxime	76	24	33	1.4
Faropenem	4	16	26	1.6
Imipenem	22	19	37	1.9
Meropenem	21	18	38	2.1
Ertapenem	8	18	38	2.1

Table S6. Hydrogen bond interactions observed for the deacylating water over adaptive string method (ASM) simulations

Acceptor Atom	Donor	Trajectory (%)	
		**Δ1**	**Δ2**
Asn170 OD1	DW	64.86	74.8
Glu166 OE2	DW	51.54	75.41
Glu166 OE1	DW	22.63	5.81
DW	Asn 170 ND2	6.12	0.16
DW	Lys73 NZ	5.24	4.54
	**Total %**	**150.39**	**160.72**

Table S7. Active site distances in molecular dynamics (MD), umbrella sampling (US) and adaptive string method (ASM) calculations

		S70-oxyanion carbonyl	T237-oxyanion carbonyl	166-170	132-hydroxyethyl oxygen
**MD**	**(Δ1)**	3.05 (0.28)	2.91 (0.13)	5.44 (1.07)	3.28 (0.64)
	**(Δ2)**	2.86 (0.18)	2.9 (0.24)	5.63 (0.88)	3.34 (0.54)
**US**	**(Δ1)**	2.74 (0.07)	2.84 (0.15)	4.85 (0.15)	3.04 (0.23)
	**(Δ2)**	2.79 (0.09)	2.9 (0.14)	4.72 (0.19)	3.32 (0.52)
**ASM**	**(Δ1)**	2.83 (0.24)	2.85 (0.15)	4.97 (0.22)	3.87 (0.46)
	**(Δ2)**	2.92 (0.31)	2.89 (0.14)	4.82 (0.26)	3.7 (0.29)

Standard deviations are shown in parentheses

Table S8 Solvent accessible surface area (SASA) in Å^2 for the KPC-2 active site during KPC-2:meropenem simulations

Simulation	Δ2	Δ1
1.5 ns MD	1416.9 (44.3)	1464.1 (76.4)
900 ps QM/MM	1454.3 (56.0)	1408.0 (23.5)
US calculations	1413 (34.4)	1400 (26.1)

Standard deviations are shown in parentheses

Figures

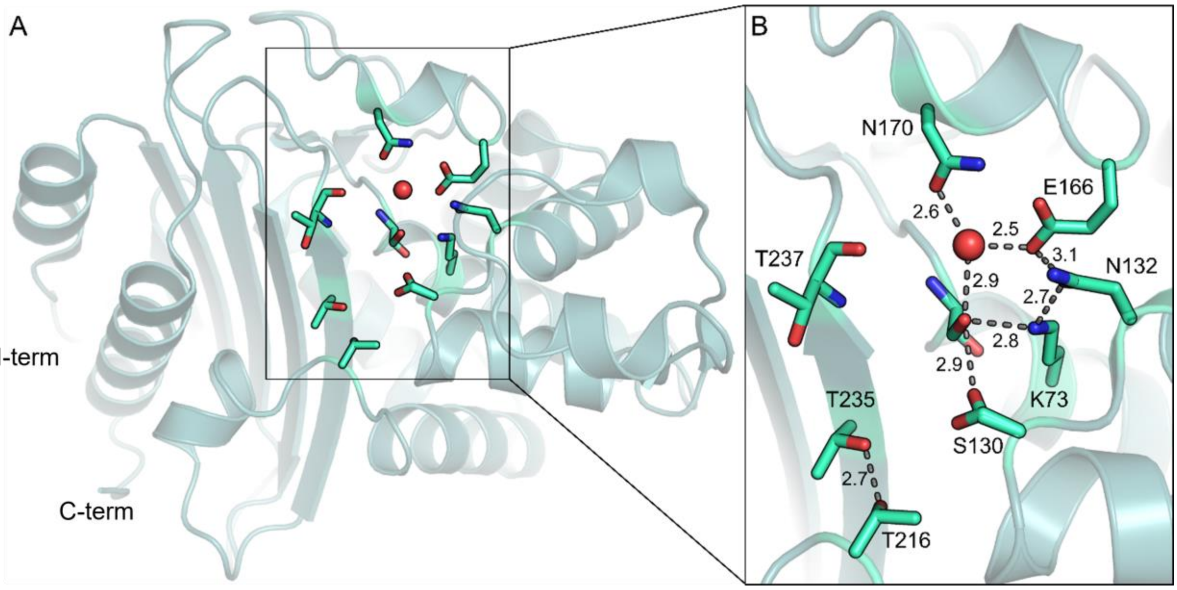

Figure S1. View of the KPC-2 apo-enzyme active site. A. Overall structure of KPC-2 (PDB ID 5UL8[1]) shown as teal cartoon with key active site residues as green-cyan sticks. **B.** Zoom in of the KPC-2 active site with key active site residues labelled and apo-enzyme hydrogen bonding networks ≤3.1 Å shown as grey dashes. Networks between residues S70, K73, S130, N132, E166, N170 and the deacylating water (DW, red sphere) are largely conserved across class A serine-β-lactamases.

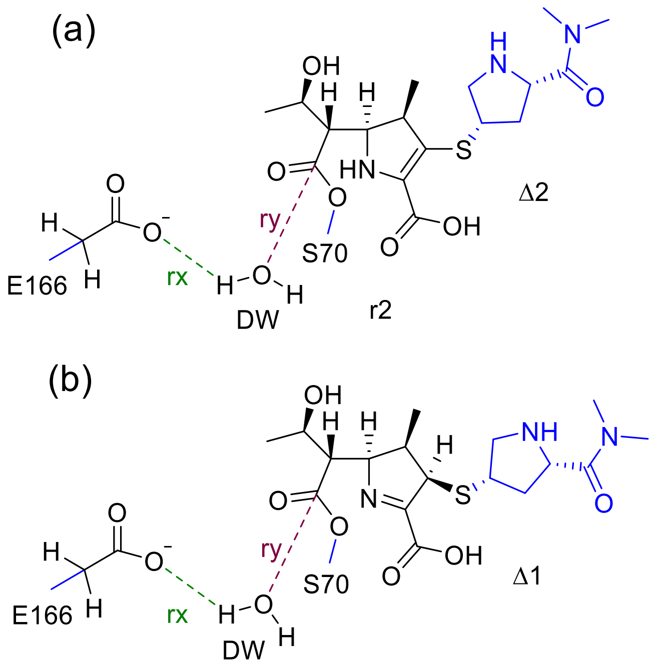

Figure S2. QM region (black) used for QM/MM calculations. Acyl-enzyme complexes of **(a)** Δ2 meropenem and **(b)** Δ1-(2*R*) meropenem. The distances between the transferred proton of the deacylating water (DW) and the Glu166 side chain (reaction coordinate 1: rx = d(OεGlu166–HDW) – d(HDW–ODW)) and the distance between the oxygen of the DW and meropenem-derived C-7 carbon (reaction coordinate 2: ry = d(C-7meropenem–ODW)) are labelled in green and red respectively. The collective variables used for the ASM were the distance between the Glu166 side chain and the closest proton of the deacylating water (collective variable 1: cv1 = d(OεGlu166–HDW) (shown in green) and the distance between the oxygen of the DW and meropenem derived C-7 carbon (collective variable 2: cv2 = d(C-7meropenem-ODW) (shown in red).

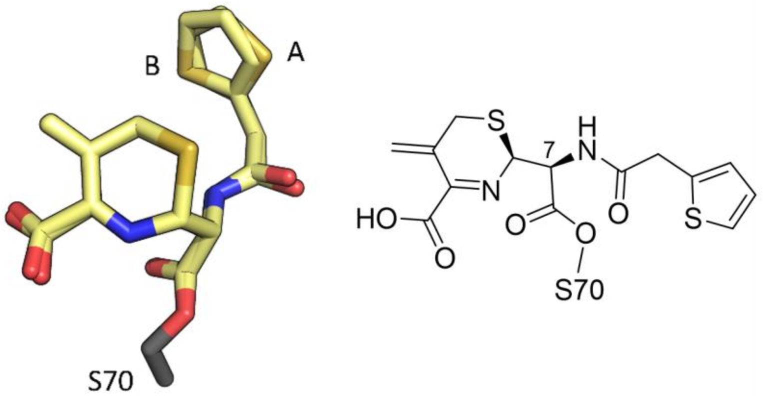

Figure S3. Evidence for two conformations of the thiophenylacetamido C-7 substituent in the KPC-2^{E166Q}:cefalothin complex. Cefalothin is shown as yellow sticks with the two orientations of the C-7 thiophenylacetamido group labelled as A and B with occupancies of 0.09 and 0.91 respectively, as defined after refinement in Phenix.

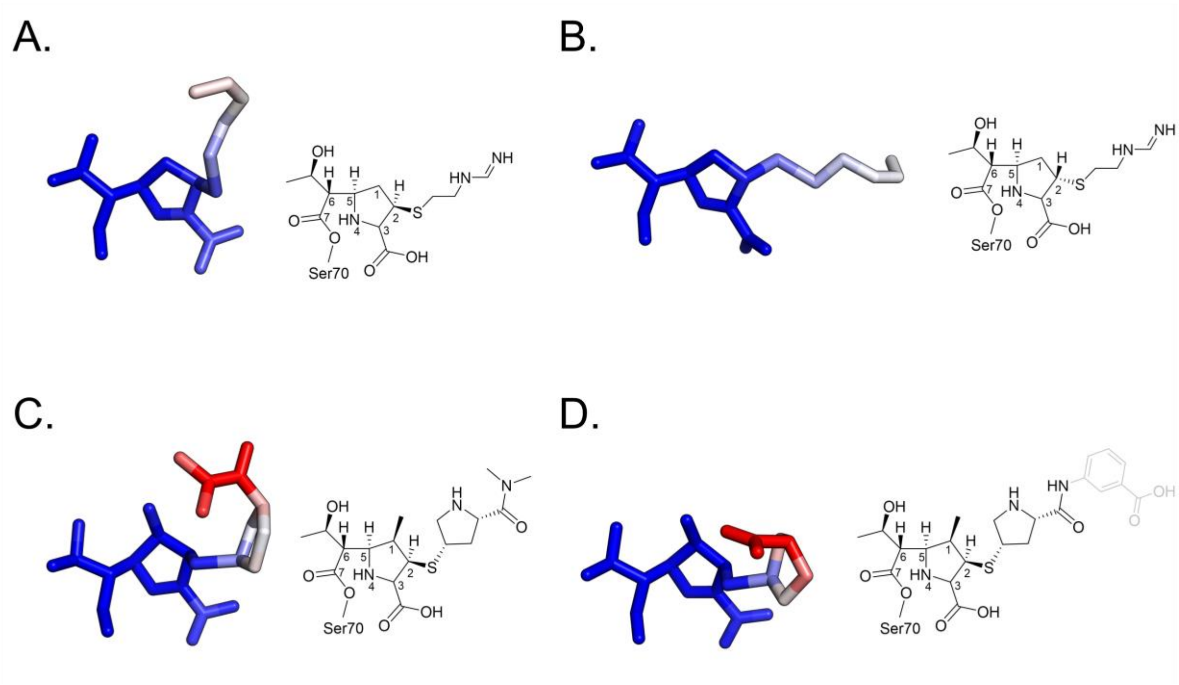

Figure S4. Carbapenem-derived acyl-enzyme complexes colored with respect to atomic B-factor. **A** Imipenem acyl-enzyme complex conformation A (Δ1-2*R*). **B.** Imipenem acyl-enzyme complex conformation B (Δ1-2*S*). **C.** Meropenem acyl-enzyme complex. **D.** Ertapenem acyl-enzyme complex. Atoms highlighted in grey could not be confidently modelled into the experimental electron density, indicating mobility.

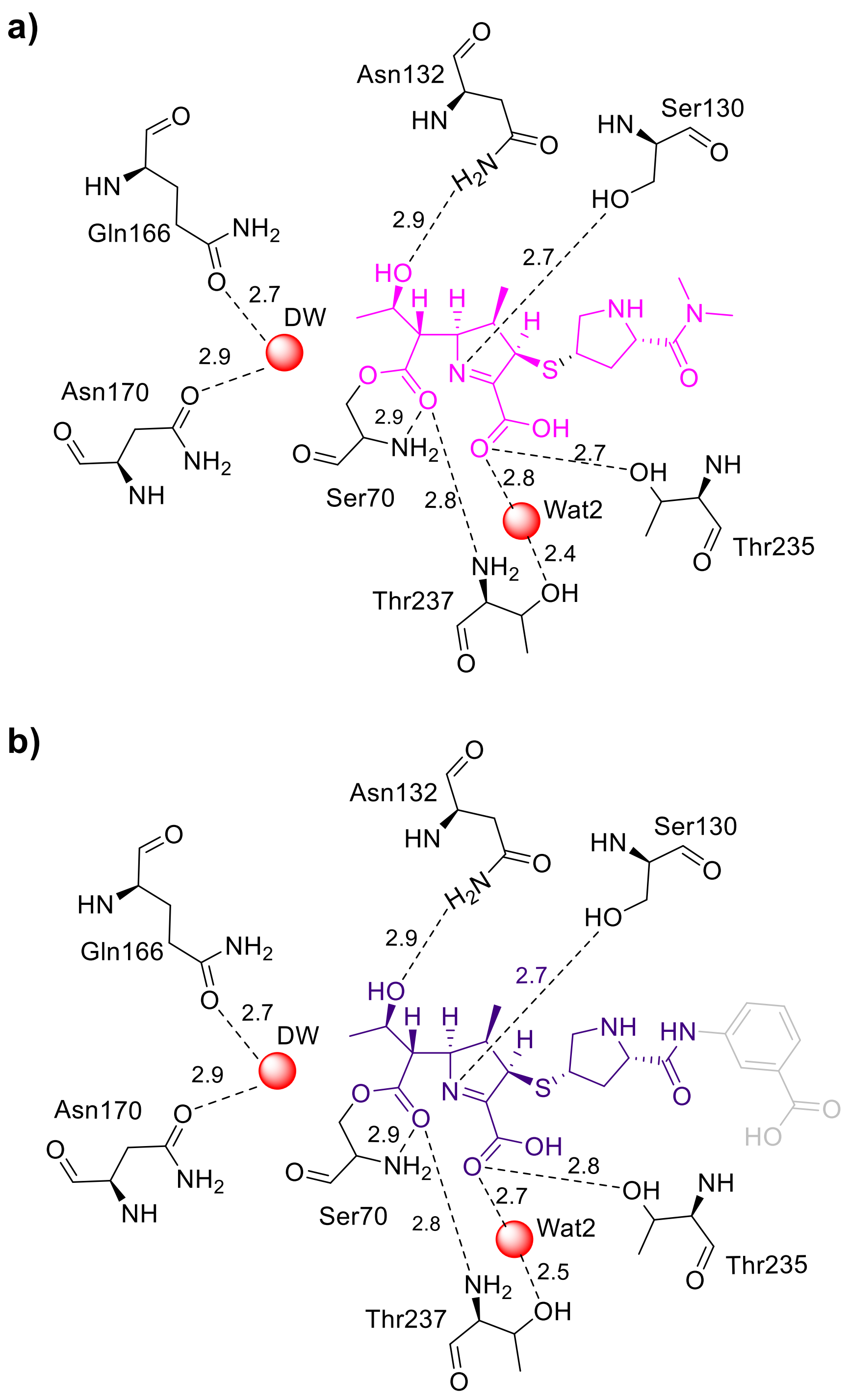

Figure S5. Possible hydrogen bonding interactions in KPC-2^{E166Q} carbapenem acyl-enzyme complexes. a) KPC-2^{E166Q}:meropenem acyl-enzyme complex, **b)** KPC-2^{E166Q}:ertapenem acyl-enzyme complex.

c)

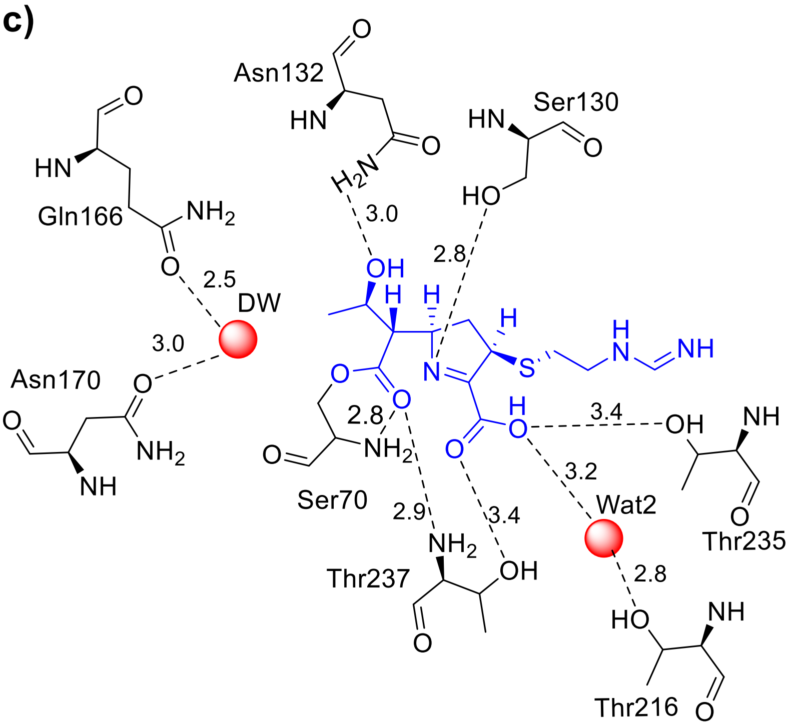

d)

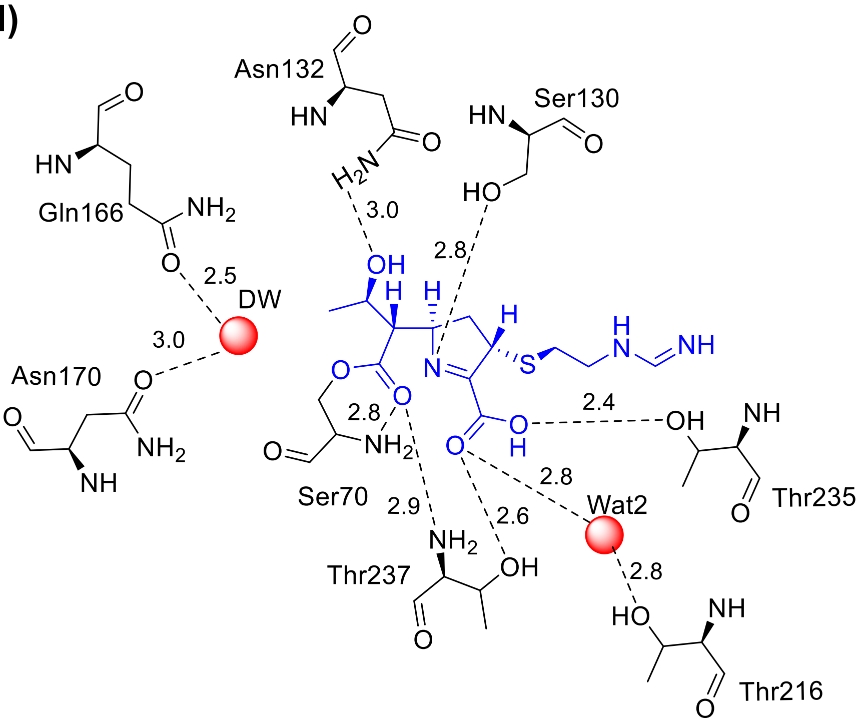

Figure S6. Possible hydrogen bonding interactions in KPC-2^{E166Q} imipenem acyl-enzyme complexes. **c)** KPC-2^{E166Q}:imipenem (conf A, (2*R*)-configuration) acyl-enzyme complex and **d)** KPC-2^{E166Q}:imipenem (conf B, (2*S*)-configuration) acyl-enzyme complex.

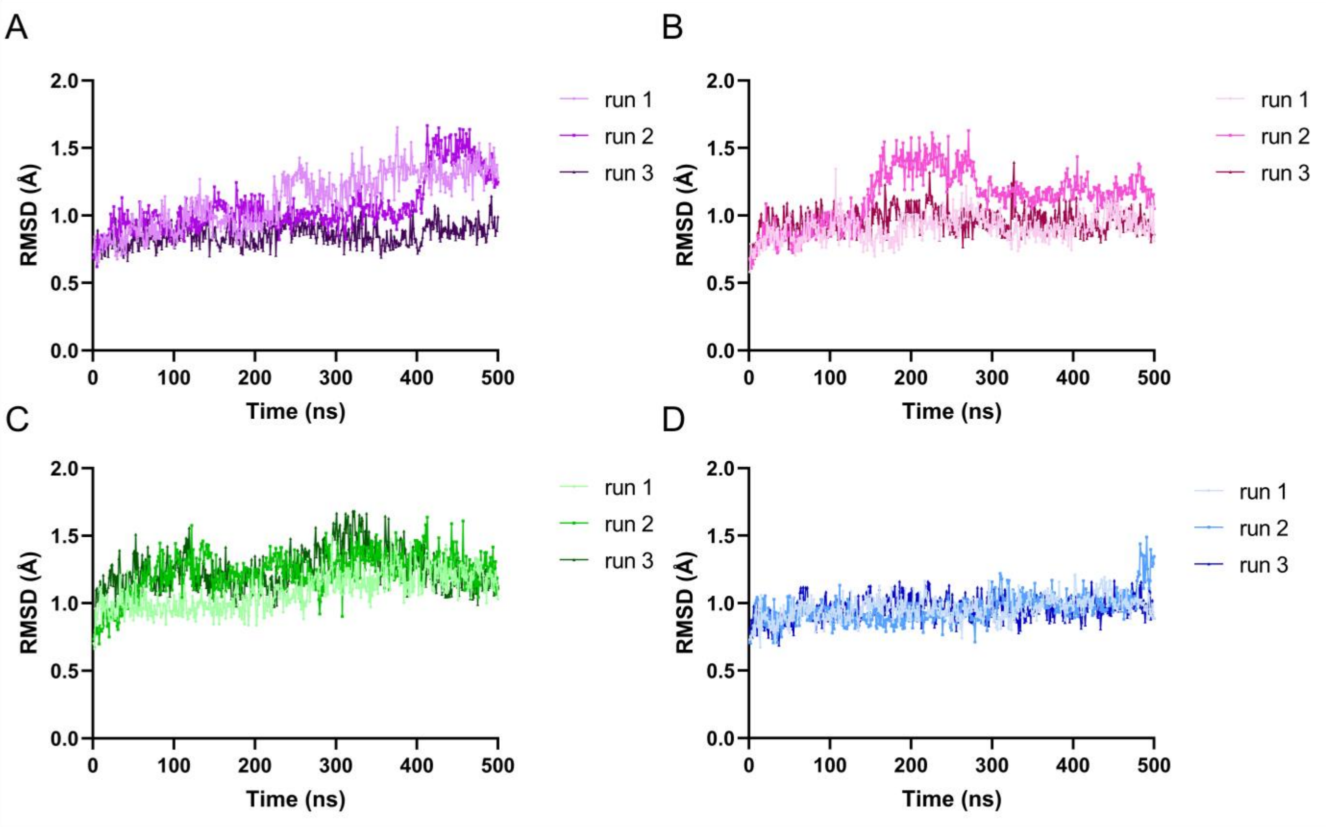

Figure S7. RMSD plots for the KPC-2:meropenem complex over triplicate 500ns simulations A. KPC-2^{E166Q}:meropenem (Δ1-(2*R*) tautomer), purple. **B.** KPC-2:meropenem (Δ1-(2*R*) tautomer), pink. **C.** KPC-2^{E166Q}:meropenem (Δ2 tautomer), green. **D.** KPC-2:meropenem (Δ2 tautomer), blue.

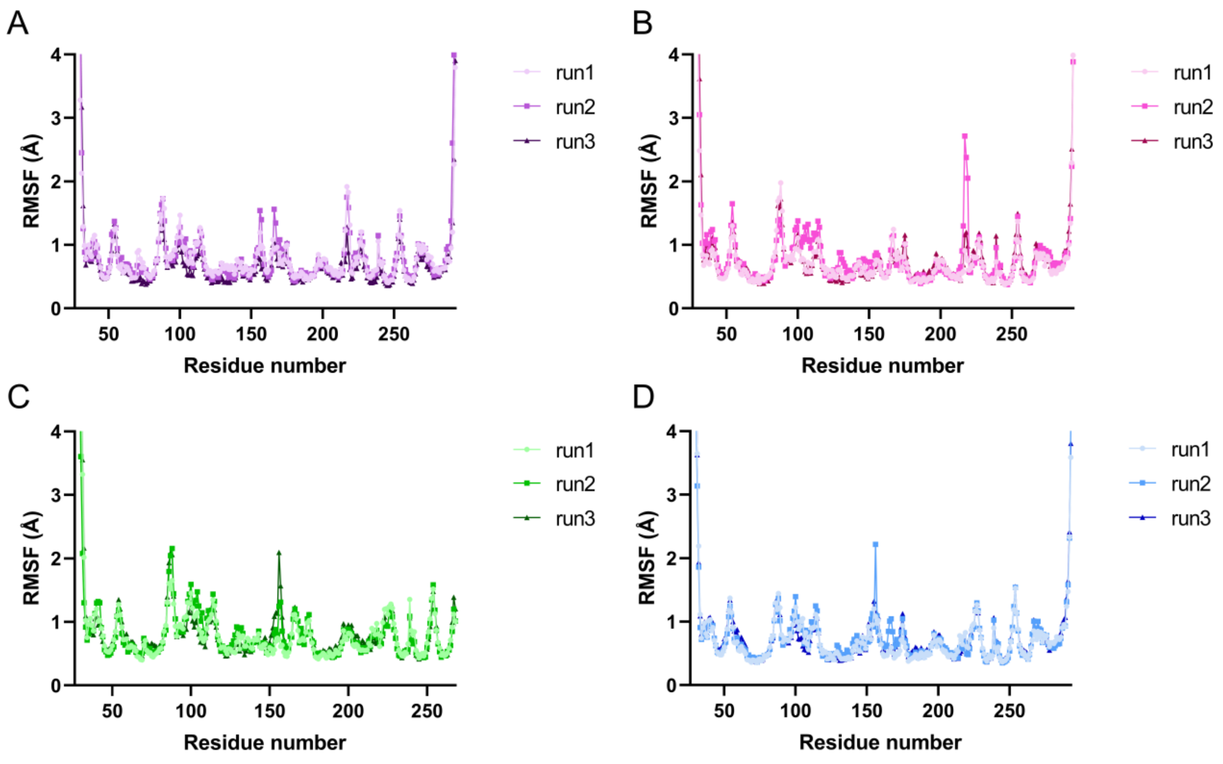

Figure S8. Per residue RMSF plots for the KPC-2:meropenem-derived acyl-enzyme complex over triplicate 500 ns simulations. A. KPC-2^{E166Q}:meropenem-derived acyl-enzyme (Δ2 tautomer) **B.** KPC-2:meropenem-derived acyl-enzyme (Δ2 tautomer) **C.** KPC-2^{E166Q}:meropenem-derived acyl-enzyme (Δ1-(2*R*) tautomer) **D.** KPC-2:meropenem-derived acyl-enzyme (Δ1-(2*R*) tautomer).

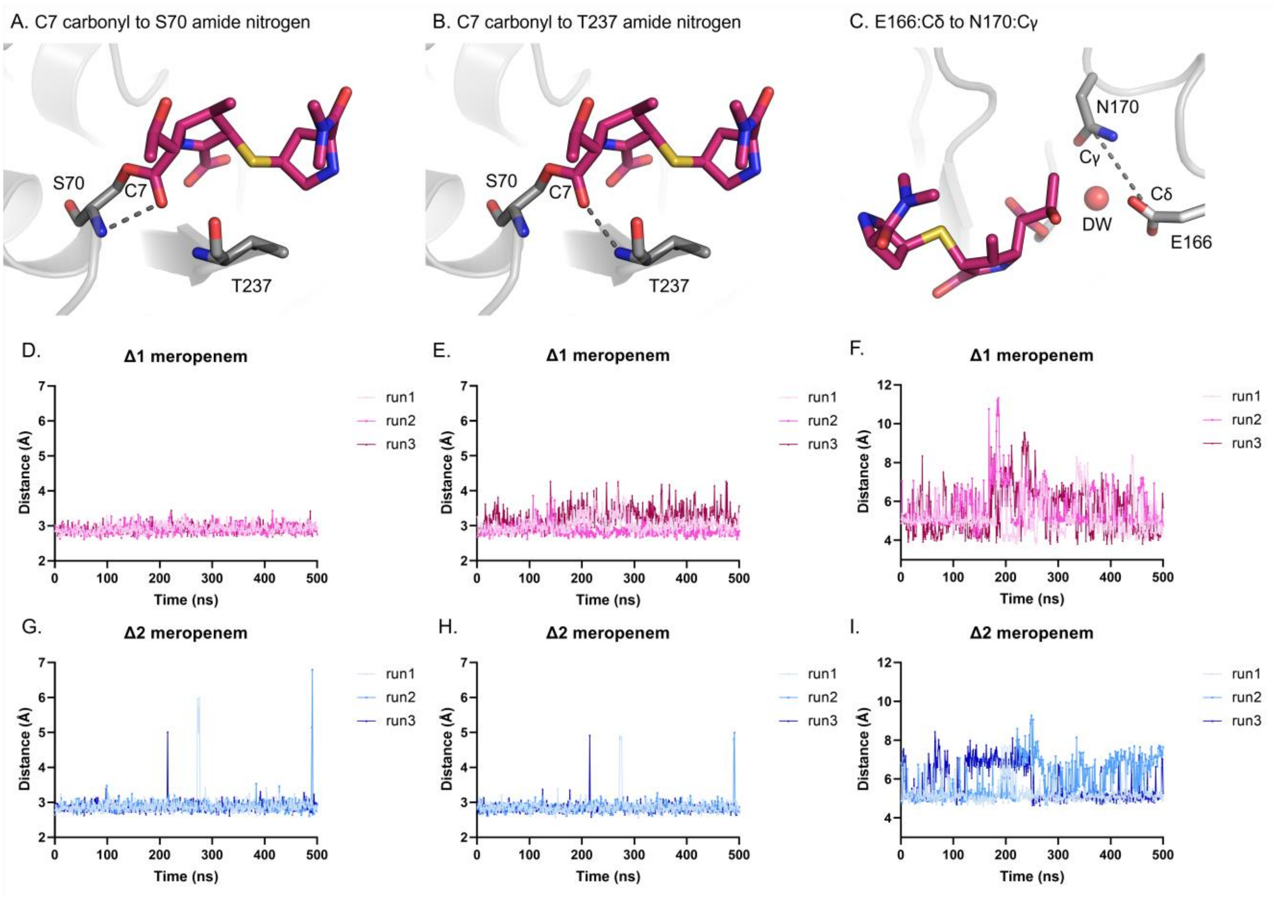

Figure S9. Distance analyses of interactions of KPC-2:meropenem-derived acyl-enzymes in triplicate MM MD simulations. A. B and **C,** starting structures of KPC-2 with the Δ1-(2*R*) meropenem-derived complex show the distances analyzed over the 500 ns trajectories. Relevant atoms and residues are labelled and the distances are represented as grey dashes. The meropenem-derived acyl-enzyme is shown as pink sticks and the KPC-2 protein as grey sticks and cartoon. The deacylating water (DW) is represented by a red sphere. **A, D** and **G,** Ser70 nitrogen (in the oxyanion hole) to the meropenem-derived C7 carbonyl distance. **B, E** and **H,** Thr237 nitrogen (oxyanion hole) to the meropenem-derived C7 carbonyl. **C, F** and **I,** distance between Cδ of Glu166 and Cγ of Asn170. **D, E** and **F,** respective distances over 500 ns trajectories between atoms in the Δ1-(2*R*) meropenem-derived acyl-enzyme shown in A, B and C. **G, H** and **I**: respective distances over 500 ns trajectories between atoms in the Δ2 meropenem-derived acyl-enzyme shown in A, B and C.

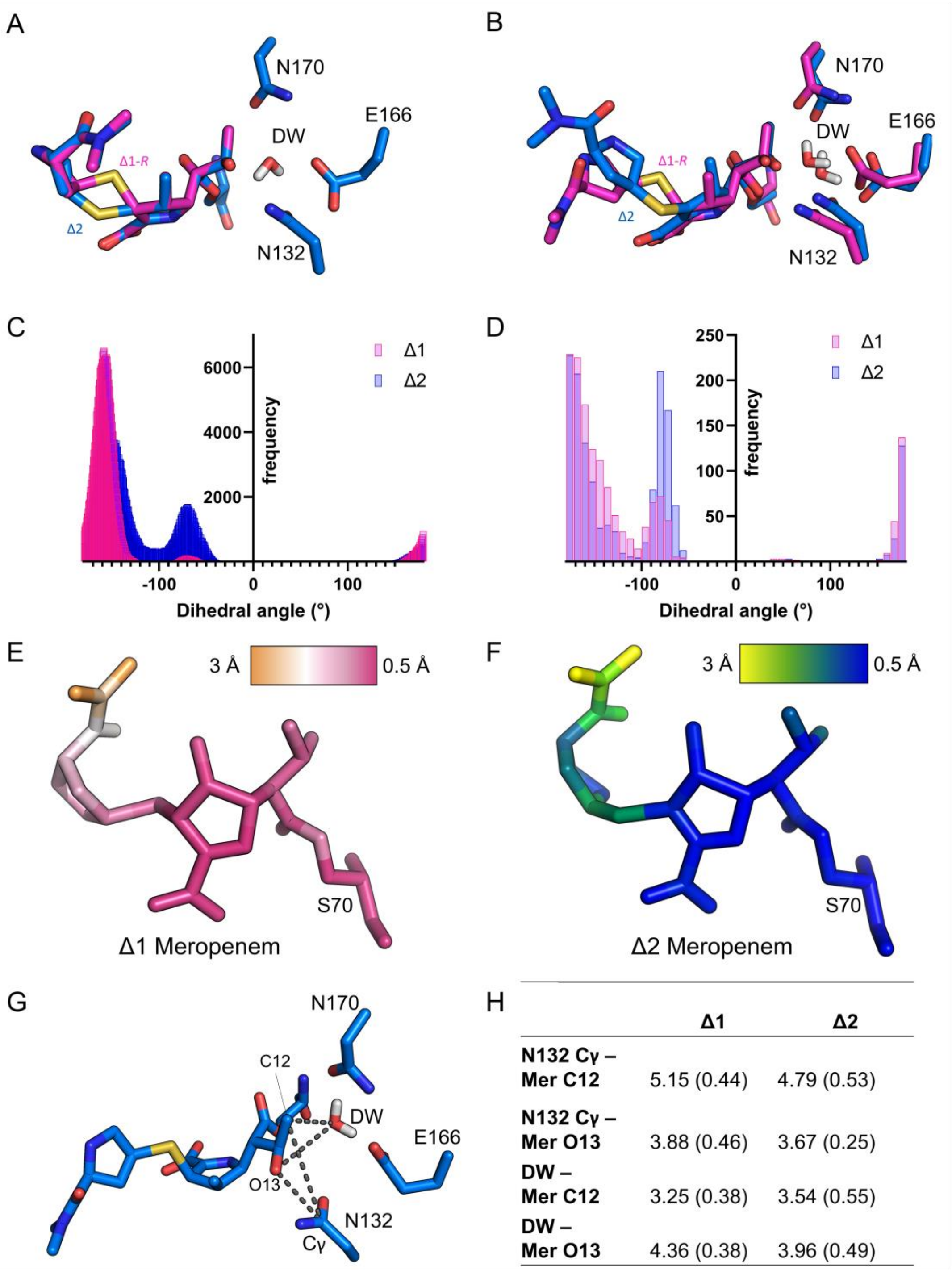

	Δ1	Δ2
**N132 Cγ – Mer C12**	5.15 (0.44)	4.79 (0.53)
**N132 Cγ – Mer O13**	3.88 (0.46)	3.67 (0.25)
**DW – Mer C12**	3.25 (0.38)	3.54 (0.55)
**DW – Mer O13**	4.36 (0.38)	3.96 (0.49)

Figure S10. Position of the meropenem C-6α hydroxyethyl group in MM and QM simulations. A. Minimized starting structures. **B.** Equilibrated starting structures (following 1 ns MM MD). Δ1-(2*R*) meropenem shown in pink, Δ2 meropenem in blue. Histograms of the hydroxyethyl dihedral angle (between atoms C-7, C-6 and the methyl carbon of the C-6 hydroxyethyl) in **C.** Triplicate Adaptive string method (ASM) simulations and **D**. Triplicate molecular dynamics simulations. Y axes represent frequency (numbers of frames). For ASM this included all sampled frames, for MD this included one frame for every ns of simulation. **E.** Ligand RMSF in ASM calculations shown on Δ1-(2*R*) meropenem (color ramped from orange to pink). **F**. Ligand RMSF in ASM calculations shown on Δ2 meropenem (color ramped from yellow to blue). **G.** Pymol representation of the distances (shown as dashes on the KPC-2:Δ2meropenem structure) between the 6α-hydroxyethyl of meropenem and the deacylating water (DW) and Asn132 in ASM simulations. **H**. Table of distances represented in **panel G** in Å.

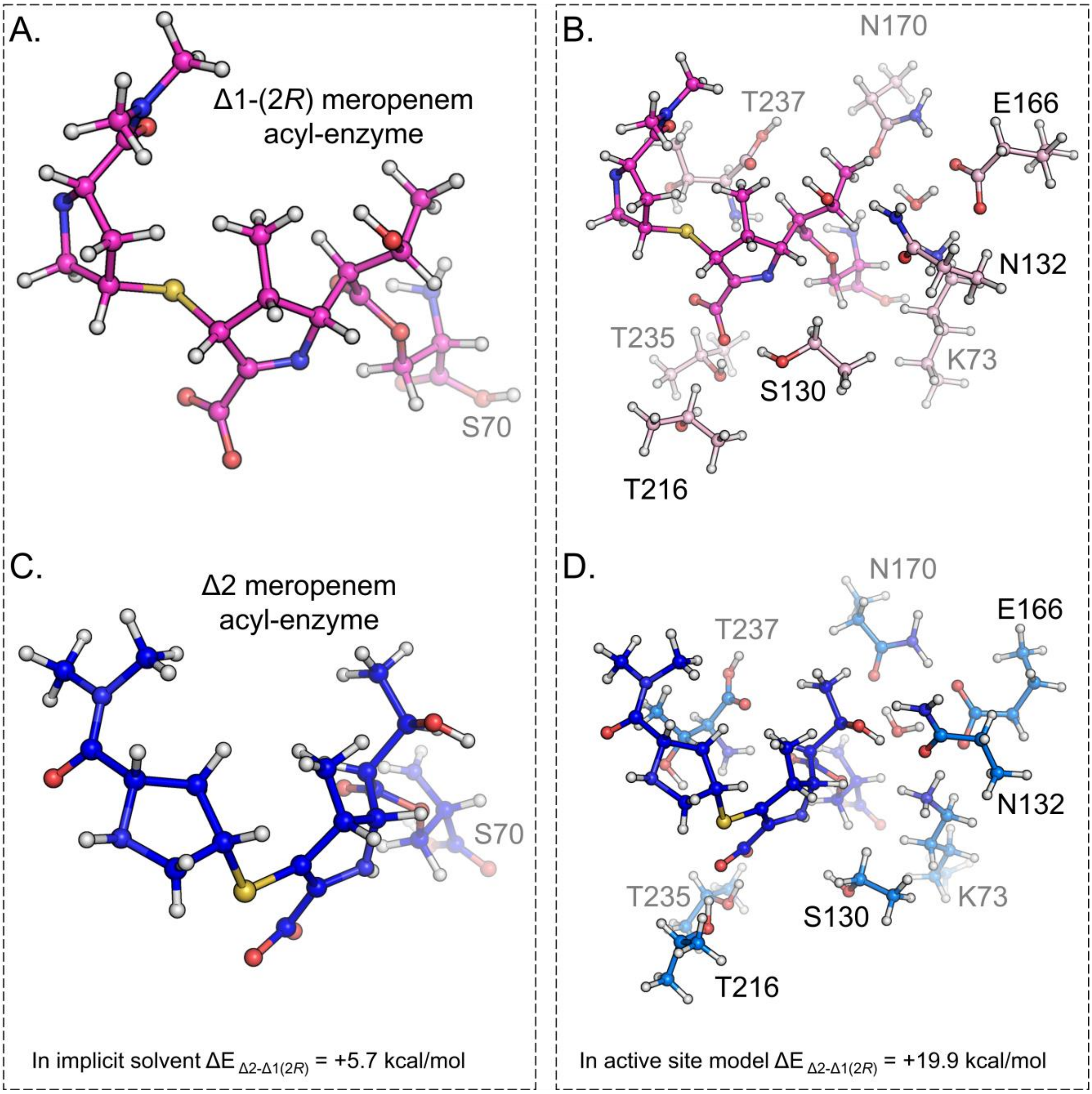

Figure S11. DFT calculations of meropenem acyl-enzymes. Representations of Δ1-(2*R*), **A.** as acyl-enzyme and **B.** active site model. Representations of Δ2 **C.** as acyl-enzyme and **D.** active site model. The change in relative energy calculated on energy-minimized meropenem Δ1 -(2*R*) and Δ2 acyl-enzymes, geometry optimizations were calculated using the B3LYP-GD3BJ method. Calculations were performed on the ligand covalently attached to Ser70 in implicit solvent (**A** and **C**) and with an active site model (**B** and **D** additionally comprising the Cα and side chain atoms of the residues K73, S130, N132, E166, N170, T216, T235 and all atoms of T237, as the backbone amide contributes to forming the oxyanion hole, and the deacylating water). A dielectric constant ε=78.4 was used when the model contained ligand covalently attached to Ser70 only with implicit solvent (panels **A** and **C**), whilst for the active site model (**B** and **D**) a dielectric constant of ε=4 was used. Relative energy differences Δ2-Δ1(2*R*) were +5.7 kcal/mol (in implicit solvent) and +19.9 kcal/mol (in active site models).

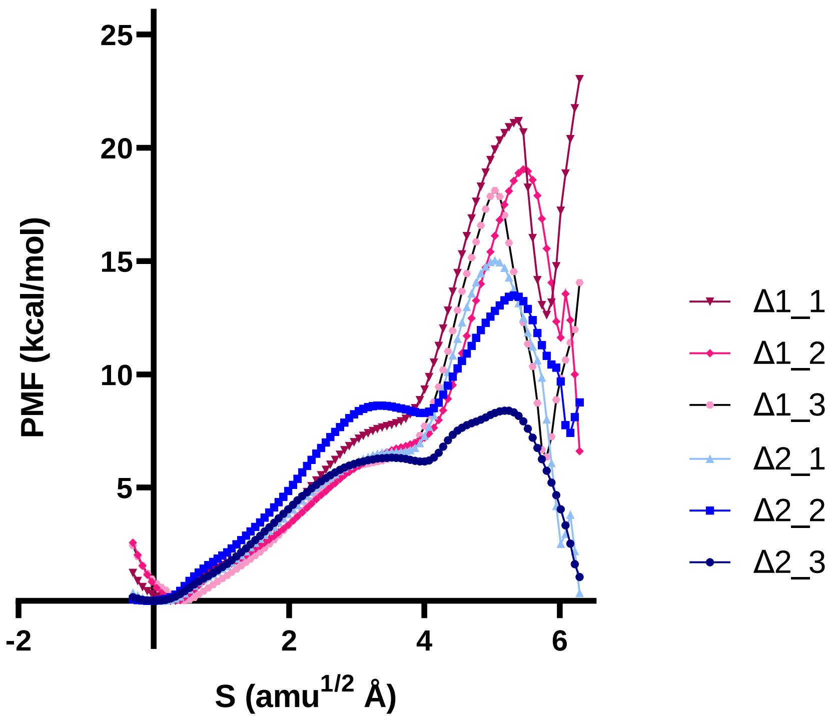

Figure S12. ASM calculations for tetrahedral intermediate formation from the meropenem-derived acyl-enzyme in KPC-2 catalysis. Potential of mean force (PMF) calculated by the ASM in kcal/mol is measured along the MFEP trajectory. Triplicate runs of each tautomer are coloured as pink (Δ1-(2*R*)) and blue (Δ2). The transition state (TS) is described as the high energy saddle point through the MFEP calculation before formation of the tetrahedral intermediate (TI). Each replicate trajectory finds its own MFEP as the adaptive string method does not employ user defined reaction coordinates, instead sampling and verifying different reaction coordinates during the string optimisation phase. This, in addition to the different starting structures used, leads to more variability than would be expected in standard umbrella sampling simulations.

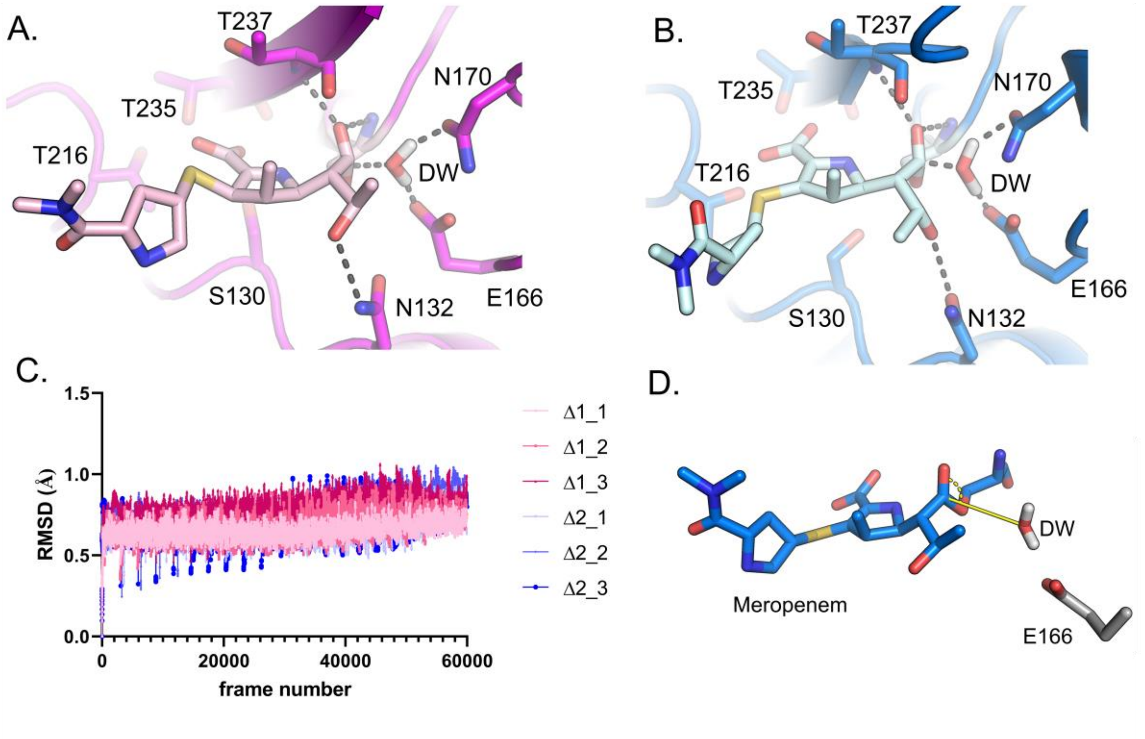

Figure S13. Transition state analysis of adaptive string method calculations. Snapshots of transition states with hydrogen bonding networks involving N132, E166, N170, T237 and the deacylating water (DW) shown as grey dashes. **A.** Δ1-(2*R*) meropenem-derived acyl-enzyme and **B.** Δ2 meropenem-derived acyl-enzyme**. C.** Acyl-enzyme RMSD during the adaptive string method (ASM) triplicate trajectories. **D.** The deacylating water (DW) to C7 carbonyl angle was measured during the ASM simulations. The average DW-C7 angle sampled in Δ1-(2*R*) meropenem simulations was 112.5°±3.4 and for Δ2 111.7°±3.6, yet Δ2 sampled the favored 107°-109° Burgi-Dunitz angle in 3.2% more frames (Δ2= 23.8% vs Δ1=20.6%) over the ASM trajectories.

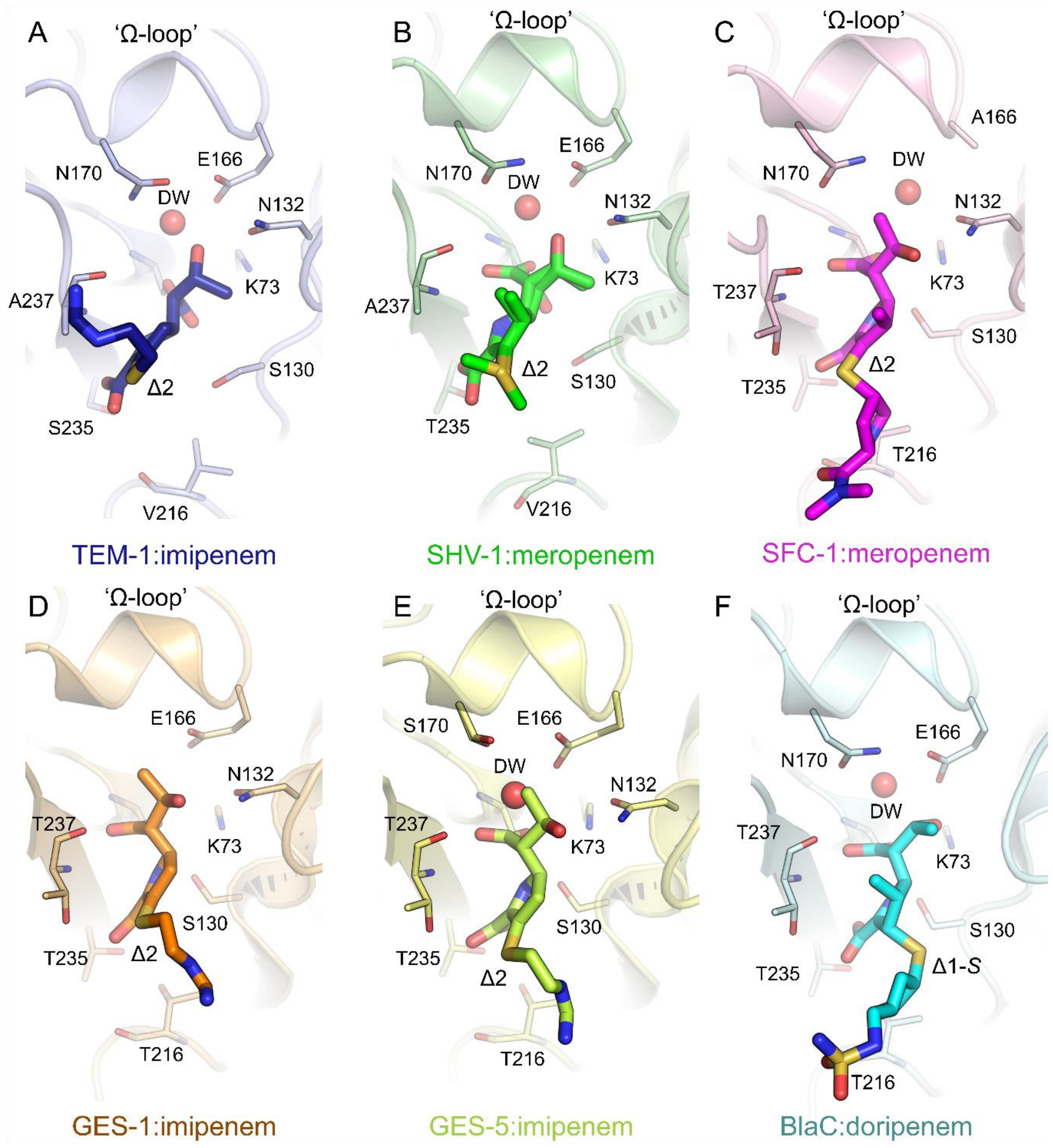

Figure S14. Comparisons of the interactions of carbapenem acyl-enzyme complexes within Class A SBLs. A. TEM-1:imipenem-derived acyl-enzyme complex (PDB:1BT5)[2], blue. **B.** SHV-1:meropenem-derived acyl-enzyme complex (PDB:2ZD8)[3], green. **C.** SFC-1:meropenem-derived acyl-enzyme complex, pink (featuring A166 mutation PDB:4EV4))[4]. **D.** GES-1:imipenem-derived acyl-enzyme complex (PDB:4GOG)[5], orange. **E.** GES-5:imipenem-derived acyl-enzyme complex (PDB:4H8R)[5], lime. **F.** BlaC:doripenem-derived acyl-enzyme complex (PDB:3IQA)[6], cyan. Carbapenem-derived acyl-enzymes are highlighted as bold sticks and key active site amino acids (if present in the structure) are labelled. The tautomeric state (Δ1-(2*R*) or Δ2) of the carbapenem-derived acyl-enzyme is labelled adjacent to the C2-sulphur. Waters closest to the putative deacylating position (if present) are shown as red spheres and labelled DW.

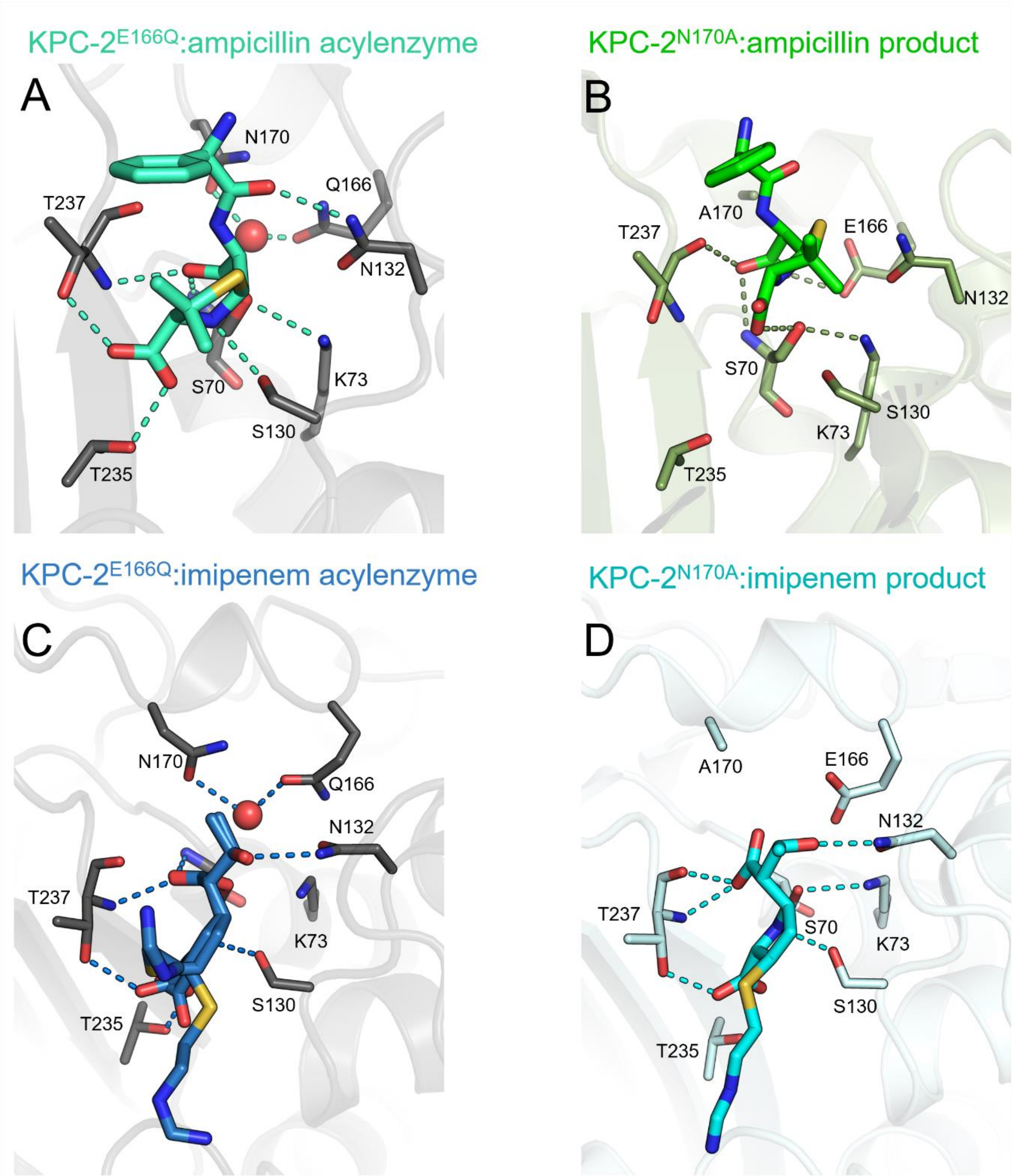

Figure S15. Comparison of KPC-2:antibiotic acyl-enzyme and non-covalent hydrolysis product complexes. Antibiotics and important active site amino acids are highlighted as sticks, possible H-bonds are identified as dashes. **A.** KPC-2^{E166Q}:ampicillin acyl-enzyme complex (teal). **B.** KPC-2^{N170A}:ampicillin non-covalent hydrolysis product (green, PDB:6XD7)[7]. **C.** KPC-2^{E166Q}:imipenem acyl-enzyme complex (blue). Note the two conformations of the carboxylate in this case. **D.** KPC-2^{N170A}:imipenem non covalent hydrolysis product (cyan, PDB 6XJ8)[7]. Note, mutation of Asn170 to Ala results in loss of a water in the deacylating position.

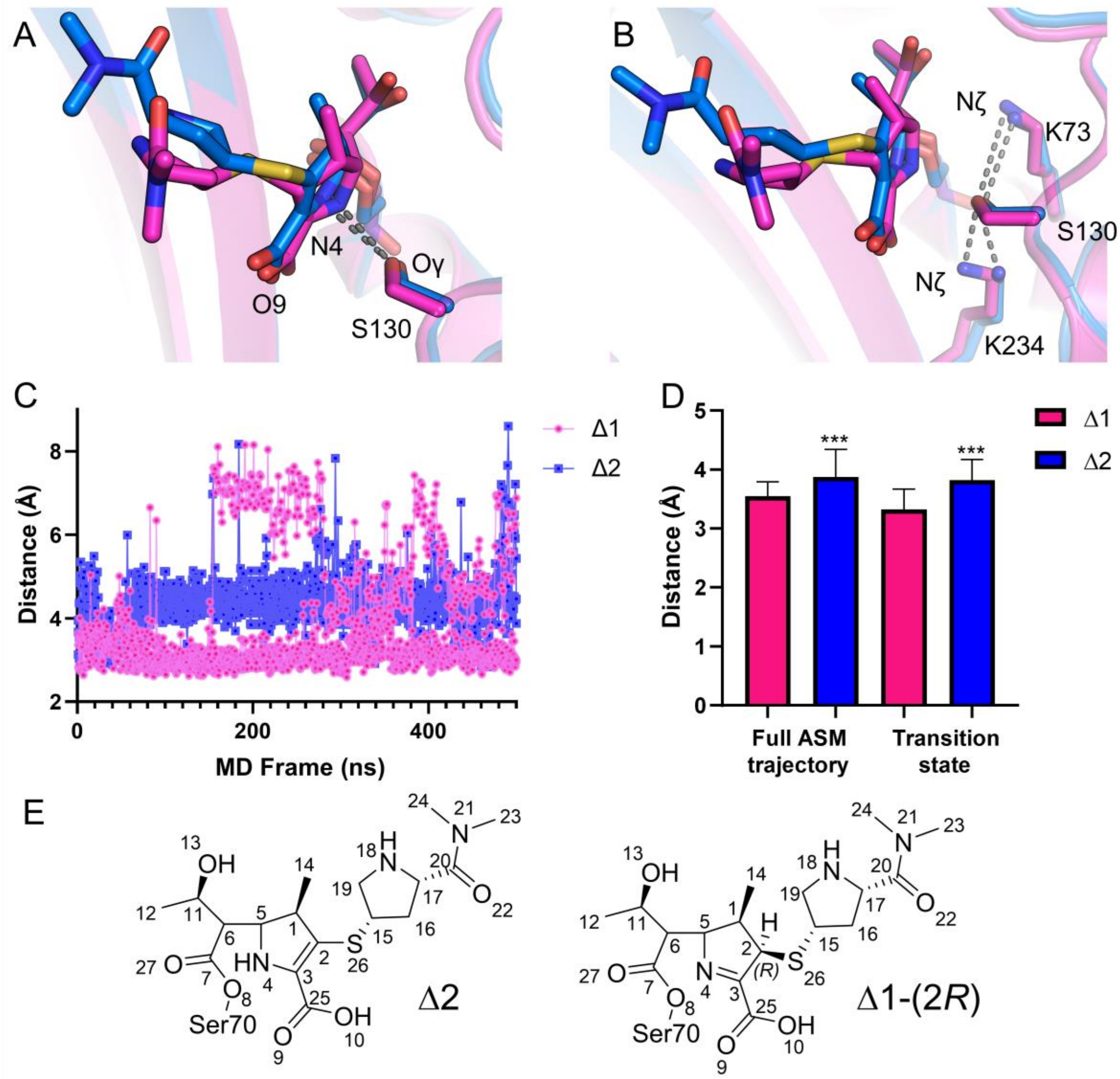

F

Acceptor	Donor	Δ1 Full Traj %	Δ2 Full Traj %	Δ1 TS %	Δ2 TS %
Ser130 Oγ	Lys234 Nζ	56.09	26.92	42.99	11.55
Ser130 Oγ	Lys73 Nζ	7.77	10.5	10.46	9.52
Mero N4	Ser130 Oγ	2.27	0.09	6.88	0.1
Mero O9	Ser130 Oγ	95.95	-	87.36	-

Figure S16. Ser130 interactions throughout MD and ASM simulations. A and B. Equilibrated starting structures (following 1 ns MM MD) Δ1-(2*R*), pink and Δ2, blue showing S130 Oγ interactions with N4 of meropenem and Nζ of Lys73 and Lys 234 respectively. C. Distance analysis of S130 to N4 in triplicate 500ns MD simulations, average distances are Δ1-(2*R*) =3.76 (±1.27) Å and Δ2=4.38 (0.59). D. Distance between meropenem N-4 and Ser130-Oγ in ASM simulations. Significance determined with unpaired t-test performed in GraphPad Prism**,** a P value of < 0.0001. E. Δ1-(2*R*) and Δ2 acyl-enzyme representations with numbered atoms corresponding to panel F. F. Hydrogen bonding interactions with Ser130 during ASM simulations for the full trajectory compared to the transition state.

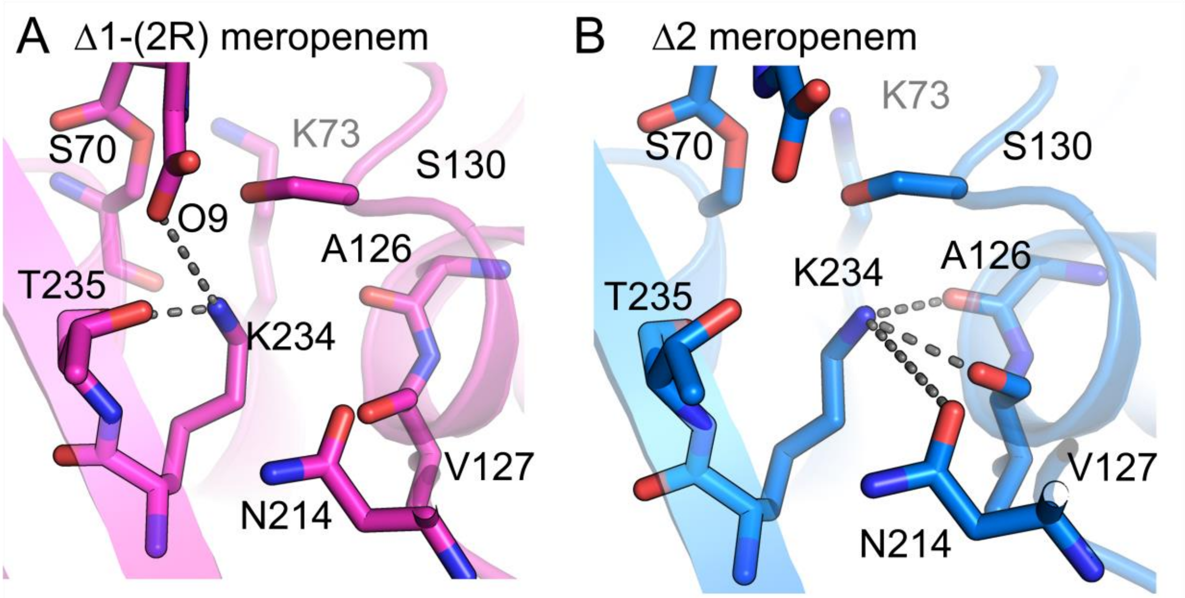

C

Acceptor	Donor	Δ1 Full Traj %	Δ2 Full Traj %	Δ1 TS %	Δ2 TS %
Val127 O	Lys234 Nζ	0	74.26	0	66.85
Asn214 Oδ1	Lys234 Nζ	0	16.40	0	0
Ala126 O	Lys234 Nζ	0	15.56	0	31.37
Thr235 O	Lys234 Nζ	79.75	0	85.55	0
Mer O9	Lys234 Nζ	5.27	0	6.74	0

Figure S17. Lys234 networks throughout ASM simulations. Representations of the distinct hydrogen bonding networks to K234 in ASM simulations **A**. Δ1-(2*R*) meropenem acyl-enzyme (equilibrated starting structure) and **B**. Δ2 meropenem acyl-enzyme (equilibrated starting structure). **C**. Distinct hydrogen bonding networks from K234, represented by % of frames.

Supporting note 1

Four systems (KPC-2^{E166Q}:meropenem-Δ2, KPC-2:meropenem-Δ2, KPC-2^{E166Q}:meropenem- Δ1-(2*R*) and KPC-2:meropenem- Δ1-(2*R*)) were energy minimised and equilibrated prior to 500 ns triplicate simulations (1.5 µs total) and analysed in cpptraj. Backbone (C_α) root mean square deviation (RMSD) values of residues 25-291 remained below 1.7 Å throughout 500 ns trajectories (**Figure S6**). The N- and C-termini displayed significant mobility in RMSD and root mean square fluctuation (RMSF) calculations (**Figures S6 and S7**) consistent with ours and others' apoenzyme simulations and analyses of KPC-2[1-2]. Small differences in RMSF around residues 216 and 217 (the 'hinge region') were visible between simulations of the Δ1-(2*R*) and Δ2 tautomer (**Figures 5 and S7**) which upon visual inspection appeared mobile throughout the duration of MD simulations[2]. Ω-Loop RMSF remained below 1.5 Å and stable in the presence of meropenem, unlike that observed for the 'bulky' oxyimino cephalosporin ceftazidime.[2] This is consistent with the experimental electron density for this region within the corresponding crystal structures (**Table S1**).

Distance measurements between backbone amide atoms (Ser70:N and Thr237:N) and the C7 carbonyl of the meropenem-derived acyl-enzyme in both Δ1-(2*R*) and Δ2 tautomers over the triplicate 500 ns MD simulations revealed these distances (and subsequent likely hydrogen bonding interactions) remained relatively stable at around 3 Å (**Figure S8**). With the Δ2 meropenem-derived acyl-enzyme, the C7 carbonyl lost interaction with the oxyanion hole in 2 frames, representing a rotation of this region in a similar orientation to that seen in crystal structures of SHV-1 and TEM-1.[3-4] This movement may also link to recent studies of KPC-2 that indicated permissive and non-permissive states may be possible during carbapenem hydrolysis.[5] Extensive analysis of Molecular Dynamics trajectories reveals no large differences in behaviour of the KPC-2:meropenem acyl-enzyme between the Δ1-(2*R*) and Δ2 tautomers. These results were expected due to the minute differences in protonation state of C3 and N4 of the carbapenem-derived scaffold (**Figure 3**).

From visual inspection of the MD trajectories, water molecules in the putative deacylating position were found to exchange frequently (between the 100ps and –5ns time scale within the simulations). However, this site remained highly occupied, with a water molecule almost always within H-bonding distance of Glu166 and Asn170, and roughly 3.5-4.5 Å from meropenem C-7.

Higher level computational approaches (combined Quantum mechanics/ Molecular mechanics, QM/MM) were therefore utilised to elucidate differences which may exist between the tautomers of the meropenem-derived acyl-enzymes. Hybrid QM/MM umbrella sampling simulations were used to investigate meropenem hydrolysis in the KPC-2 acyl-enzyme.

In a protocol that we have previously shown to be effective for modeling deacylation of carbapenem-derived acyl-enzymes by class A β-lactamases[12], we employ the SCC-DFTB2[13-15] QM method in the AMBER16 simulation package,[16] using the ff14SB[17] MM forcefield for protein, the TIP3P-Ew[18] water model and the General AMBER Force Field (GAFF)[16] for meropenem-derived regions not in the QM region. The QM region (modelled with a charge of -2) comprises the water in the deacylating position (DW), Ser70, Glu166 and the common carbapenem scaffold. Conventional QM/MM umbrella sampling MD was performed along two distance-based reaction coordinates to simulate the deacylation reaction (**Figure S5**). The first is the linear combination of distances between the distance of Glu166 OE2 and the nearest proton of the deacylating water and the distance between the same proton and the oxygen of the deacylating water, 1: rx = d(OεGlu166–HDW) – d(HDW–ODW) which corresponds to proton transfer from DW to Glu166. The second is the distance from the newly formed hydroxide to the C8 carbonyl, 2: = d(C7meropenem–ODW) which represents the nucleophilic attack of DW on the carbonyl carbon (**Figure S5**). 20 ps of QM/MM MD were performed for each simulation window, and simulations repeated in triplicate. 2D minimum free energy paths (MFEPs) were calculated as previously described[12], using the weighted-histogram analysis method (WHAM); the highest point (saddle point) along the MFEP is taken as the transition state, giving the activation free energy, $\Delta^{\ddagger}G_{\text{calc}}$ **Table S4**). The adaptive string method (ASM) path calculation method was also used for MFEP calculation of the first step of carbapenem deacylation. Two collective variables were used with the ASM, with the first collective variable subtly differing to the first reaction coordinate used for

conventional US: The distance between Glu166 OE2 and a proton of the deacylating water, 1: rx = d(OεGlu166–HDW) and the distance between the meropenem C7 and the oxygen of the newly formed hydroxide, 2: ry = d(C7meropenem–ODW). Each node along the string was sampled for 60 ps and the simulations repeated in triplicate.

Supporting note 2

We measured the Ser130-Oγ to N4 distance over ASM trajectories derived from QM/MM MD simulations of the respective acyl-enzymes, finding significant differences both across the whole trajectory and between the respective deacylation transition states (**Figure S16**). Analysis of H-bond networks determined that Ser130 is likely positioned, by Lys73 and Lys234, to interact with either the meropenem C3 carboxylate or N4 over both the whole trajectory and the transition state frames. Indeed, further analysis of the orientation of Ser130 throughout the ASM trajectories also reveals more consistent hydrogen bonding to the Lys234 side chain amide (Lys234-NZ) in the Δ1-(2*R*) (imine) tautomer than in the Δ2 (enamine) (**Figure S15**). Thus, more stable electrostatic interactions in the Δ1-(2*R*) acyl-enzyme sustain Ser130-Oγ in a position close to either meropenem-O9 or meropenem N-4, whilst the relative instability of the Δ2 acyl-enzyme allows the Ser130 side chain to explore conformational space further from meropenem N-4. These differences directly result from differences between tautomers in the hydrogen bonding network around the Lys234 side chain (**Figure S17**). Specifically, Lys234-NZ makes interactions exclusive to each tautomer; hydrogen bonding predominantly to the Thr235 backbone oxygen in the Δ1-(2*R*), and to the Val127 backbone oxygen in the Δ2 configuration (**Figure S17**). In consequence different orientations of the Lys234 side chain prevail in each tautomer, subsequently hindering its propensity to hydrogen bond to Ser130-Oγ in Δ2 but promoting it in Δ1-(2*R*), accounting for the difference between tautomers in the distance between Ser130-Oγ and meropenem N-4. Alongside the H-bond networks around T216, T235 (as described in results) our simulations reveal that the Δ1-(2*R*) meropenem acyl-enzyme makes more extensive hydrogen bonding interactions with KPC-2, and is consequently more stable, than the Δ2, resulting in an increased free energy barrier to tetrahedral intermediate formation, as evidenced by the 7.1 kcal/mol difference between tautomers identified by ASM calculations.

Supporting Information References

1. Pemberton, O. A.; Zhang, X.; Chen, Y., Molecular Basis of Substrate Recognition and Product Release by the Klebsiella pneumoniae Carbapenemase (KPC-2). *J Med Chem* **2017,** *60* (8), 3525-3530.
2. Maveyraud, L.; Mourey, L.; Kotra, L. P.; Pedelacq, J.-D.; Guillet, V.; Mobashery, S.; Samama, J.-P., Structural Basis for Clinical Longevity of Carbapenem Antibiotics in the Face of Challenge by the Common Class A β-Lactamases from the Antibiotic-Resistant Bacteria. *Journal of the American Chemical Society* **1998,** *120* (38), 9748-9752.
3. Nukaga, M.; Bethel, C. R.; Thomson, J. M.; Hujer, A. M.; Distler, A.; Anderson, V. E.; Knox, J. R.; Bonomo, R. A., Inhibition of class A beta-lactamases by carbapenems: crystallographic observation of two conformations of meropenem in SHV-1. *J Am Chem Soc* **2008,** *130* (38), 12656-62.
4. Fonseca, F.; Chudyk, E. I.; van der Kamp, M. W.; Correia, A.; Mulholland, A. J.; Spencer, J., The Basis for Carbapenem Hydrolysis by Class A β-Lactamases: A Combined Investigation using Crystallography and Simulations. *Journal of the American Chemical Society* **2012,** *134* (44), 18275-18285.
5. Smith, C. A.; Frase, H.; Toth, M.; Kumarasiri, M.; Wiafe, K.; Munoz, J.; Mobashery, S.; Vakulenko, S. B., Structural Basis for Progression toward the Carbapenemase Activity in the GES Family of β-Lactamases. *Journal of the American Chemical Society* **2012,** *134* (48), 19512-19515.
6. Tremblay, L. W.; Fan, F.; Blanchard, J. S., Biochemical and structural characterization of Mycobacterium tuberculosis beta-lactamase with the carbapenems ertapenem and doripenem. *Biochemistry* **2010,** *49* (17), 3766-73.
7. Mehta, S. C.; Furey, I. M.; Pemberton, O. A.; Boragine, D. M.; Chen, Y.; Palzkill, T., KPC-2 β-lactamase enables carbapenem antibiotic resistance through fast deacylation of the covalent intermediate. *The Journal of biological chemistry* **2021,** *296*, 100155.
8. Galdadas, I.; Qu, S.; Oliveira, A. S. F.; Olehnovics, E.; Mack, A. R.; Mojica, M. F.; Agarwal, P. K.; Tooke, C. L.; Gervasio, F. L.; Spencer, J.; Bonomo, R. A.; Mulholland, A. J.; Haider, S., Allosteric communication in class A β-lactamases occurs via cooperative coupling of loop dynamics. *eLife* **2021,** *10*, e66567.
9. Tooke, C. L.; Hinchliffe, P.; Bonomo, R. A.; Schofield, C. J.; Mulholland, A. J.; Spencer, J., Natural variants modify Klebsiella pneumoniae carbapenemase (KPC) acyl-enzyme conformational dynamics to extend antibiotic resistance. *The Journal of biological chemistry* **2021,** *296*, 100126.
10. Nukaga, M.; Bethel, C. R.; Thomson, J. M.; Hujer, A. M.; Distler, A.; Anderson, V. E.; Knox, J. R.; Bonomo, R. A., Inhibition of Class A β-Lactamases by Carbapenems: Crystallographic Observation of Two Conformations of Meropenem in SHV-1. *Journal of the American Chemical Society* **2008,** *130* (38), 12656-12662.
11. Cortina, G. A.; Hays, J. M.; Kasson, P. M., Conformational Intermediate That Controls KPC-2 Catalysis and Beta-Lactam Drug Resistance. *ACS Catalysis* **2018,** *8* (4), 2741-2747.
12. Chudyk, E. I.; Limb, M. A. L.; Jones, C.; Spencer, J.; van der Kamp, M. W.; Mulholland, A. J., QM/MM simulations as an assay for carbapenemase activity in class A β-lactamases. *Chemical Communications* **2014,** *50* (94), 14736-14739.
13. de M Seabra, G.; Walker, R. C.; Elstner, M.; Case, D. A.; Roitberg, A. E., Implementation of the SCC-DFTB method for hybrid QM/MM simulations within the amber molecular dynamics package. *J Phys Chem A* **2007,** *111* (26), 5655-64.
14. Woodcock, H. L.; Hodoscek, M.; Brooks, B. R., Exploring SCC-DFTB paths for mapping QM/MM reaction mechanisms. *J Phys Chem A* **2007,** *111* (26), 5720-8.
15. Elstner, M.; Seifert, G., Density functional tight binding. **2014,** *372* (2011), 20120483.
16. D.A. Case, J. T. B., R.M. Betz, D.S. Cerutti, T.E. Cheatham, III, T.A. Darden, R.E. Duke, T.J. Giese, H. Gohlke, A.W. Goetz, N. Homeyer, S. Izadi, P. Janowski, J. Kaus, A. Kovalenko, T.S. Lee, S. LeGrand, P. Li, T. Luchko, R. Luo, B. Madej, K.M. Merz, G. Monard, P. Needham, H. Nguyen, H.T. Nguyen, I. Omelyan, A. Onufriev, D.R. Roe, A. Roitberg, R. Salomon-Ferrer, C.L. Simmerling, W. Smith, J. Swails, R.C. Walker, J. Wang, R.M. Wolf, X. Wu, D.M. York and P.A. Kollman, AMBER 2015. University of California, San francisco, 2015.

17. Maier, J. A.; Martinez, C.; Kasavajhala, K.; Wickstrom, L.; Hauser, K. E.; Simmerling, C., ff14SB: Improving the Accuracy of Protein Side Chain and Backbone Parameters from ff99SB. *J Chem Theory Comput* **2015,** *11* (8), 3696-713.
18. Jorgensen, W. L.; Chandrasekhar, J.; Madura, J. D.; Impey, R. W.; Klein, M. L., Comparison of simple potential functions for simulating liquid water. *The Journal of Chemical Physics* **1983,** *79* (2), 926-935.
